# Supplementary material for: OPtimising Treatment for MIld Systolic hypertension in the Elderly (OPTiMISE): protocol for a randomised controlled non-inferiority trial
Source: BMJ Open. 2018 Oct 4;8(9):e022930. doi: 10.1136/bmjopen-2018-022930 (PMC6173263; doi:10.1136/bmjopen-2018-022930)
Supplement: Author's manuscript [file bmjopen-2018-022930.draft_revisions.pdf]

BMJ Open is committed to open peer review. As part of this commitment we make the peer review history of every article we publish publicly available.

When an article is published we post the peer reviewers' comments and the authors' responses online. We also post the versions of the paper that were used during peer review. These are the versions that the peer review comments apply to.

The versions of the paper that follow are the versions that were submitted during the peer review process. They are not the versions of record or the final published versions. They should not be cited or distributed as the published version of this manuscript.

BMJ Open is an open access journal and the full, final, typeset and author-corrected version of record of the manuscript is available on our site with no access controls, subscription charges or pay-per-view fees (http://bmjopen.bmj.com).

If you have any questions on BMJ Open's open peer review process please email info.bmjopen@bmj.com

BMJ Open

OPtimising Treatment for MIld Systolic hypertension in the Elderly (OPTiMISE): protocol for a randomised controlled non-inferiority trial

Journal:	*BMJ Open*
Manuscript ID	bmjopen-2018-022930
Article Type:	Protocol
Date Submitted by the Author:	13-Mar-2018
Complete List of Authors:	Sheppard, James; University of Oxford, Nuffield Department of Primary Care Health Sciences<br>Burt, Jenni; University of Cambridge, Cambridge Centre for Health Services Research<br>`Lown, Mark; University of Southampton<br>Temple, Eleanor; University of Oxford, Nuffield Department of Primary Care Health Sciences<br>Benson, John; University of Cambridge, GP and Primary Care Research Unit<br>Ford, Gary; Oxford University Hospitals NHS Foundation Trust, Oxford Academic Health Science Network<br>Heneghan, Carl; Oxford University, Primary Health Care<br>Hobbs, Richard; University of Oxford, Nuffield Department of Primary Care Health Sciences<br>Jowett, Sue; University of Birmingham, Health Economics Unit<br>Little, Paul; University of Southampton, Primary Care and Population Science;<br>Mant, Jonathan; University of Cambridge, General Practice and Primary Care Research Unit<br>Mollison, Jill; University of Oxford, Nuffield Department of Primary Care Health Sciences<br>Nickless, Alecia; University of Oxford, Nuffield Department of Primary Care Health Sciences<br>Ogburn, Emma; University of Oxford,<br>Payne, Rupert; University of Bristol, Centre for Academic Primary Care<br>Williams, Marney; Patient and public involvement representative<br>Yu, Ly-Mee; University of Oxford, Department of Primary Care Health Sciences<br>McManus, Richard; University of Oxford, Dept of Primary Care Health Sciences
Keywords:	Multi-morbidity, Cardiovascular disease, Frailty, Antihypertensive, De-prescribing
Note: The following files were submitted by the author for peer review, but cannot be converted to PDF. You must view these files (e.g. movies) online.	
OPTiMISE video inforgraphic V1.1 14.03.17.mp4	

SCHOLARONE™
Manuscripts

For peer review only

OPtimising Treatment for MIld Systolic hypertension in the Elderly (OPTiMISE): protocol for a randomised controlled non-inferiority trial

James P Sheppard,[1] Jenni Burt,[2] Mark Lown,[3] Eleanor Temple,[1] John Benson,[4] Gary A Ford,[1] Carl Heneghan,[1] FD Richard Hobbs,[1] Sue Jowett,[5] Paul Little,[3] Jonathan Mant,[4] Jill Mollison,[1] Alecia Nickless,[1] Emma Ogburn,[1] Rupert Payne,[6] Marney Williams,[7] Ly-Mee Yu,[1] and Richard J McManus[1]

[1]Nuffield Department of Primary Care Health Sciences, University of Oxford, Oxford, UK
[2]The Healthcare Improvement Studies Institute, University of Cambridge, Cambridge, UK
[3]Primary Care Research Group, University of Southampton, Southampton, UK
[4]Primary Care Unit, Department of Public Health and Primary Care, University of Cambridge, Cambridge, UK
[5]Institute of Applied Health Research, University of Birmingham, Birmingham, UK
[6]Centre for Academic Primary Care, University of Bristol, Bristol, UK
[7]Patient and public involvement representative, London, UK

Corresponding author: James P Sheppard
Email: james.sheppard@phc.ox.ac.uk
Telephone: +44 1865 617192
Address: Nuffield Department of Primary Care Health Sciences, Radcliffe Primary Care Building, Radcliffe Observatory Quarter, University of Oxford, Oxford, OX2 6GG, UK

Trial Sponsor: University of Oxford
Contact name: Ms Heather House
Address: Clinical Trials and Research Governance, Joint Research Office, Block 60, Churchill Hospital, University of Oxford, Oxford, OX3 7LE
Email: ctrg@admin.ox.ac.uk

Word count: 4,208 (excluding title page, abstract, references, tables and figures)
Number of tables: 2
Number of figures: 3

For peer review only

Abstract

Introduction: Recent evidence suggests that larger blood pressure reductions and multiple antihypertensive drugs may be harmful in older people, particularly frail individuals with polypharmacy and multi-morbidity. However, there is a lack of evidence to support de-prescribing of antihypertensives, which limits the practice of medication reduction in routine clinical care. The aim of this trial is to examine whether antihypertensive medication reduction is possible in older patients without significant changes in blood pressure control at follow-up.

Methods and analysis: This trial will use a Primary Care based, open label, randomised controlled trial design. A total of 540 participants will be recruited, aged ≥80 years, with systolic blood pressure <150 mmHg and receiving ≥2 antihypertensive medications. Participants will have no compelling indication for medication continuation and will be considered to potentially benefit from medication reduction due to existing polypharmacy, co-morbidity and frailty. Following a baseline appointment, individuals will be randomised to a strategy of medication reduction (intervention) with optional self-monitoring or usual care (control). Those in the intervention group will have one antihypertensive medication stopped. The primary outcome will be to determine if a reduction in medication can achieve a proportion of participants with clinically safe blood pressure levels at 12 week follow-up (defined as a systolic blood pressure <150mmHg) which is non-inferior (within 10%) to that achieved by the usual care group. Qualitative interviews will be used to understand the barriers and facilitators to medication reduction. The study will use economic modelling to predict the long term effects of any observed changes in blood pressure and quality-of-life.

Ethics and dissemination: The protocol and written information has been approved by a Research Ethics Committee, medicines regulatory authority (MHRA), and national and local health research authorities. All research outputs will be published in peer-reviewed journals and presented at national and international conferences.

Trial registration: EudraCT 2016-004236-38

Word count: 299 (max 300)

Keywords: Multi-morbidity, cardiovascular disease, frailty, antihypertensive, de-prescribing

For peer review only

For peer review only - http://bmjopen.bmj.com/site/about/guidelines.xhtml

Strengths and weaknesses of this study

- This will be the first UK randomised controlled trial to compare a strategy of antihypertensive medication reduction to usual care in primary care.
- The pragmatic trial design, with broad inclusion criteria, will make findings of the study externally valid in routine clinical practice.
- Allowing the attending GP to choose the medication to be reduced will maximise external validity of the trial results but precludes the possibility of blinding the participants and investigators.
- The trial will be powered to detect a non-inferior difference in blood pressure control at follow-up, but not necessarily secondary outcomes such as differences in rates of cardiovascular disease, adverse events and quality of life.

For peer review only

For peer review only - http://bmjopen.bmj.com/site/about/guidelines.xhtml

Introduction

The general population is ageing[1] and, consequently, the number of people living with age-related chronic conditions is increasing.[2] Hypertension is the number one co-morbid condition in older people with multiple chronic conditions[3] and 52% of those aged ≥80 years are prescribed two or more antihypertensive drugs (equivalent to approximately 1.25 million people in the UK).[4] Blood pressure lowering has been shown to be effective at preventing stroke and cardiovascular disease in healthy individuals aged ≥80 years with stage 2 hypertension (systolic blood pressure of >160mmHg) and high risk stage 1 hypertension.[5 6] However, as with many trials,[7 8] these studies included healthier populations with lower polypharmacy and multi-morbidity than might be expected in the general elderly population. In addition, there is evidence to suggest that larger blood pressure reductions and multiple antihypertensive prescriptions may be harmful in older people.[9 10] Evidence from observational studies also suggests that higher intensity blood pressure treatment is associated with increased risk of falls in older people,[11] although this is also disputed.[5]

Some patients consider the increased risk of falls and other adverse events to be as important as the risk of myocardial infarction or stroke, particularly those taking medications for primary prevention of cardiovascular disease.[12] Thus, decisions over blood pressure lowering in the elderly, particularly the frail elderly, require the weighing of harms and quality of life. However, clinicians can often struggle to stop prescribing medication due to a perceived lack of evidence, fear of the reaction of other prescribers, fear of precipitating events such as stroke or angina and concern that patients will feel their care is being cut.[13 14]

There is limited evidence from randomised trials examining the safety of antihypertensive medication reduction or withdrawal.[15-19] The HYVET trial[5] enrolled some patients on antihypertensive treatment who were then randomised to placebo (effectively complete medication withdrawal), but there are few trials comparing a specified strategy of antihypertensive medication reduction with usual care in terms of effects on blood pressure control and quality of life.[17] In addition, there are no previous economic modelling studies of a strategy of medication reduction in the elderly.

The aim of this work will be to examine whether antihypertensive medication reduction in patients with controlled systolic hypertension (<u><</u>150mmHg) who are being prescribed two or more antihypertensives is possible without significant changes in blood pressure control at follow-up.

Methods

Aims and outcomes

The aim of this study is to determine whether antihypertensive medications can be safely reduced without systolic blood pressure increasing beyond clinically safe levels at follow-up.

The primary outcome is the proportion of participants with clinically safe levels at 12 week follow-up (defined as a systolic blood pressure <150mmHg) Secondary outcomes will examine:

- The proportion of participants in the intervention arm who maintain medication reduction through to follow-up (*i.e.* are *not* restarted on therapy).
- The difference in quality of life (according to EQ-5D-5L) between groups at 12 week follow-up.
- The difference in frailty (according to the frailty index)[20] between the two groups at 12 week follow-up.
- The difference in the change in mean clinic systolic blood pressure (from baseline) between the two groups at 12 week follow-up.
- The difference in reported side effects to medication between the two groups at 12 week follow-up (including coughs, dizziness, syncope, and ankle swelling).

For peer review only - http://bmjopen.bmj.com/site/about/guidelines.xhtml

- The difference in routinely reported serious adverse events between the two groups at 12 week follow-up (hospitalisation due to falls, myocardial infarction, stroke or all-cause mortality).

Design

This trial will use a Primary Care based, open label, randomised controlled, two-parallel groups, non-inferiority trial design, recruiting 540 participants with controlled blood pressure (systolic <150 mmHg) on two or more antihypertensive treatments. Participants will be randomised to a strategy of medication reduction (intervention) or usual care (control) and followed-up for 12 weeks (see figure 1). Embedded qualitative and economic analyses will examine barriers and facilitators to medication reduction and the cost effectiveness of the approach.

Trial participants

Patients eligible for the trial will be aged ≥80 years, with systolic blood pressure <150mmHg (current UK guideline recommendation)[21] receiving ≥2 antihypertensive medications. They will have no compelling indication for medication continuation and in the opinion of the attending GP, may potentially benefit from medication reduction due to existing polypharmacy, co-morbidity and/or frailty (table 1).

Participants will be identified and recruited from general practices via the UK Clinical Research Network (CRN). Potentially eligible patients will be identified by trained practice staff searching practice-based electronic disease registers using a standardised strategy. GPs will be asked to check the search results and remove people whom they believe to be unsuitable to participate in the study. Remaining potentially eligible patients will be sent letters of invitation from their GP and those expressing an interest in the trial will be asked to attend a screening and baseline appointment. Patients not responding to the first invitation will receive one reminder letter (up to four weeks after the first letter). Other potentially eligible patients may also be approached opportunistically by a member of the clinical care team. Those who do not wish to take part will be asked to fill in a short questionnaire detailing their reasons.

Baseline visit

Eligible patients will have informed consent taken by the GP. During the consent appointment, the GP will show a two-minute study video infographic (see supplementary material) and go through the participant information sheet explaining the exact nature of the trial. Having had a chance to ask questions, those individuals willing to participate will give written informed consent by means of a participant dated signature and dated signature of the GP who presented and obtained the informed consent.

Some participants will be invited to have their interview audio-recorded for qualitative analysis during their study visits. Those who are interested will be asked to sign a response slip prior to meeting the GP. Consent to audio recordings will not have a bearing on an individual's care or eligibility for the main trial.

Those giving informed consent will be screened using the criteria in table 1 and undergo baseline measurements by a member of the research team via participant questionnaires and a detailed notes review (table 2). Blood pressure will be measured in a standardised fashion using the clinically validated[22] BpTRU blood pressure monitor which automatically records six blood pressure measurements at one minute intervals. Blood pressure readings will be taken in the left arm (where appropriate) after participants have been seated for at least five minutes of rest, using an appropriate sized cuff. The mean of the 2nd and 3rd readings will be used to define the primary outcome. To test for orthostatic hypotension, two further readings will be taken in the standing position after one and three minutes.[23] Orthostatic

For peer review only - http://bmjopen.bmj.com/site/about/guidelines.xhtml

hypotension will be defined as a ≥20mmHg drop in systolic blood pressure within three minutes of standing.

All data will be collected via an electronic case report form (eCRF) linked to the study database. Participants will be given the option to enter responses to questionnaires themselves or with assistance from the research team. Where questionnaires are not validated for use on a tablet computer,[24] or where individuals are not comfortable using one, paper copies will be made available for completion.

Randomisation

Consenting participants will be individually randomised (1:1 allocation ratio) to one of two study arms using a fully validated web-based system (Sortition®) with manual telephone back up. Participants will not be randomised until after consent has been taken and baseline assessments have been completed. A computer generated non-deterministic algorithm, minimising on practice and baseline systolic blood pressure will be used to ensure these covariates are balanced between the two intervention arms.

The study will use an open label design, so patients and practitioners will not be blinded to the intervention or study endpoints. Therefore, codebreaking will not be necessary. The statistical analysis will be performed blind to patient allocation.

Intervention group

Participating GPs will review each participant's antihypertensive medication regimen prior to the baseline appointment, and decide which medication should be removed if they are randomised to the intervention arm of the trial. The choice of medication to be withdrawn will be at the discretion of the GP, but their decision will be informed by an individual's co-morbidities and existing guidelines, where appropriate (figure 2). Specifically, participating GPs will be encouraged to identify previously unrecognised contraindications to medication, defined by the STOPP criteria.[25] In the absence of these, or a strong clinical rationale for continuing despite a STOPP criteria being met, GPs will be recommended to reduce antihypertensive medications in reverse of the NICE C+A+D algorithm for older patients (figure 2).[21] All participants in the trial will remain on at least one antihypertensive.

Once a medication has been removed, GPs or other appropriate, delegated healthcare professionals will closely monitor the participant's response to medication reduction: they will be given advice about what and when to monitor (figure 3), but this schedule will be flexible. All participants will be expected to return for at least one routine safety follow-up visit, and further visits may be required if blood pressure is raised (≥150 mmHg), or adverse events occur. Where blood pressure is persistently raised, GPs will be expected to re-adjust medication (dose or type), rendering the likelihood of a serious adverse event occurring as a result of the intervention very low.

Self-monitoring

All participants randomised to the medication reduction arm of the trial will be given the option to self-monitor their blood pressure at home. Those accepting will be trained using protocols developed in the previous TASMIN trials[26 27] and will be given simple and clear instructions to contact their GP if their blood pressure rises above what is considered clinically safe (e.g. home systolic blood pressure >145mmHg on all readings taken in a week). Participants will be advised to self-monitor (or have a carer monitor) at least 4 times per week in the last week of each month of follow-up (weeks 4, 8 and 12), although they can monitor more frequently if they wish. Differential use of self-monitoring in the intervention group, or indeed in the

For peer review only - http://bmjopen.bmj.com/site/about/guidelines.xhtml

control group (many patients now self-monitor routinely)[28] is not expected to impact on the study results, since there is good evidence that self-monitoring only affects blood pressure levels if used in combination with a co-intervention.[29] All other clinical care will continue as usual.

Control group

Those allocated to the control arm of the study will continue usual clinical care (i.e. they will continue to take antihypertensive medications as prescribed and will not self-monitor unless already doing so). No other medication changes will be mandated and participating GPs will be asked to manage all other care according to usual clinical practice.

Follow-up visits

Participants will attend one research follow-up clinic, 12 weeks (±2 weeks) after baseline and those in the intervention will attend one additional safety visit after four weeks (±2 weeks) (figure 1). A period of four weeks is expected to be sufficiently long enough to assess the impact of antihypertensive medication reduction, since these drugs usually take approximately four weeks to 'wash out' of a patient's system. Earlier safety visits are not recommended since they could provide false reassurance that blood pressure is within safe limits if the withdrawn drug has not washed out of the participant's system.

The follow-up assessments will include standardised blood pressure measurement (for assessment of the primary outcome), questionnaire assessments and adherence to the trial medication regime, side effects and adverse events (table 2). Where possible, all participants will be flagged for mortality and hospital admissions using NHS patient tracking services, permitting long-term follow-up for up to 5 years after the trial has finished.

Each participant has the right to withdraw from the trial at any time. We will ask all participants to attend a follow-up visit as far as is practicable, regardless of whether medication is re-introduced to participants in the intervention group, or a participant in the control group has medication withdrawn. Unless a participant withdraws consent, vital status will be assessed even where an individual has been lost to follow-up (for instance moved away). If given, the reason for withdrawal will be recorded in the eCRF.

Internal feasibility study

A two-stage internal feasibility study will be conducted to examine methods of patient invitation and rates of recruitment, before proceeding with the main trial. The first feasibility phase will last for a minimum of three months and aim to recruit approximately 25 participants from a minimum of 3-5 practices. The aim will be to establish whether or not anyone will be willing to participate in the study.

The second feasibility phase will focus on recruitment rates for the main trial and whether the intended sample size is likely to be met during the recruitment period. This phase will have a recruitment target of 75 participants from ten practices over 6 months, giving a total sample for the feasibility study of 100 participants. A recruitment rate of 15% of invitations sent is expected. The following actions will be considered to address varying rates of recruitment at the end of the feasibility phases:

- If ≥100 participants are recruited – trial will proceed as planned
- If 75-99 participants are recruited – recruitment materials/method will be re-examined and edited where necessary following discussions with stakeholders and patient and public involvement representatives.

For peer review only - http://bmjopen.bmj.com/site/about/guidelines.xhtml

- If 50-74 participants are recruited – the allocation of resources and recruitment criteria will be re-examined using information gathered from concurrent qualitative work.
- If <50 participants are recruited – the Trial Steering Committee (TSC) will decide, in discussion with the Data Monitoring and Ethics Committee (DMEC) and the funders, whether the trial should be stopped due to futility.

Sample size calculation

Assuming that 100% of participants in the usual care group, and 96% of those in the medication reduction group have controlled systolic blood pressure levels (<150mmHg) at follow-up, approximately 540 participants will be required to detect a non-inferior difference in systolic blood pressure control between groups. Calculations assume a 10% non-inferiority margin, 90% power, 2.5% 1-sided level of significance, 10% loss to follow-up and a 10% dilution effect due to cross-over between arms. There is no existing precedent for an appropriate margin of non-inferiority in this type of trial, but 10% was deemed useful to inform future doctor-patient discussions about medication reduction: if the non-inferiority margin is met, it will suggest that for every ten patients who have their medication reduced, at least nine will still have controlled blood pressure at 12 week follow-up.

Statistical analysis

A detailed statistical analysis plan will be agreed prior to the end of the trial. The primary and secondary analyses will be by intention to treat (ITT), unless stated otherwise. The primary analysis will be a non-inferiority analysis by means of the "two one-sided test" (TOST) procedure,[30] whereby the 95% confidence interval for the relative risk of participants with systolic blood pressure at 12 weeks below 150 mmHg between the medication reduction group and the usual care group is calculated. This will be obtained by means of a generalised linear mixed effects model with GP surgery included as a random effect and baseline blood pressure as a fixed effect. If the lower limit of the confidence interval is more than 0.9 (equal to a risk difference of 10%) then the research hypothesis that medication reduction will be non-inferior in terms of blood pressure control to usual care will be accepted. As a secondary analysis of the primary outcome, a per-protocol (PP) analysis will be performed, since ITT can be anticonservative for a non-inferiority hypothesis.[30] Participants who received the medication reduction intervention in the PP analysis will be defined as a participant in the medication reduction arm who maintained their medication reduction throughout the 12 week follow-up period.

Secondary analyses will examine the proportion of participants in the medication reduction arm who maintained their medication reduction throughout the 12 week follow-up period. Secondary outcomes will be analysed by means of linear mixed effects models, adjusting for the baseline level of the outcome and baseline systolic blood pressure and including practice as a random effect: systolic blood pressure, EQ-5D-5L and the Frailty index/frail scale. The difference in the rate of side effects and adverse events between the medication reduction and usual care arms will be analysed by means of a logistic mixed effects model adjusting for baseline systolic blood pressure and including practice as a random effect.

Exploratory subgroup analyses of blood pressure control, change in blood pressure and maintenance of medication reduction will be conducted by different levels of baseline frailty, functional independence, cognitive function, number of medications prescribed at baseline and number of co-morbidities at baseline.

For peer review only - http://bmjopen.bmj.com/site/about/guidelines.xhtml

Patient and public involvement
This protocol was developed through discussions with older patients and carers and members of an AgeUK day centre. MW is a stroke survivor with experience as a volunteer on the elderly ward of Charing Cross Hospital. She was consulted on the suitability and design of the trial and is a member of the trial management group. Methods of patient approach, including the design of the video infographic, patient information sheet and consent form were all reviewed by patient representatives prior to formal approval. The TSC includes two independent patient representatives responsible for overseeing the conduct of the trial.

Safety reporting
Adverse events that are observed by an investigator or reported by the participant will be recorded on the AE log at any time during the study but AEs will be specifically asked about at the 12 week follow up. Serious adverse events (SAEs) will be reported to the coordinating centre within 24 hours of discovery or notification of the event. All SAE reports will be reviewed by the DMEC chair on a monthly basis, and by the full DMEC at meetings held every 6 months. The DMEC will include a geriatrician, statistician and consultant clinical pharmacologist. They will be responsible for safeguarding trial participants, monitoring emerging trial data including identifying any trends, such as increases in unexpected events, and take appropriate action where necessary.

All adverse events labelled possibly, probably or definitely related will be considered as related to the intervention. Since there are no sections of the Summary of medicinal Product Characteristics, or previous clinical studies which detail expected adverse events as a result of medication withdrawal, all SAEs at least possibly related, and not as a result of re-introduction of withdrawn medication, will be considered unexpected and reported as SUSARs. Fatal and life-threatening SUSARs will be reported by the chief investigator to the relevant Competent Authority and Research Ethics Committee no later than 7 calendar days after the Sponsor or delegate is first aware of the reaction. All other SUSARs will be reported within 15 calendar days.

Qualitative sub-studies
Study 1: interviews with doctors and patient
Face-to-face interviews with GPs and patients will be conducted to generate understanding about the barriers and facilitators to antihypertensive medication reduction. Informed consent will be sought from approximately 15 GPs to provide a broad range of opinion from varying practice sizes (small to large) and settings (rural to urban). Participating GPs will also be asked to identify up to 15 patients for interview, based on the same inclusion criteria as those applied to participants in the main trial.

Interviews with GPs will use a chart-stimulated recall approach to explore the factors which influence their treatment choices in older hypertensive patients. Anonymised electronic health records will be used to focus discussions about how GPs would feel about reducing antihypertensive medications. Interviews with patients will use 'brown bag' medication review techniques[31] to create a complete record of medication held, with a commentary on usage from the participants' perspective. Diagrammatic elicitation techniques will be used to complete a relational map outlining participants' circumstances and how these relate to the medications taken. These sketches will be used as the basis for a discussion on the implications of withdrawing antihypertensive medications, and what this "gap" might mean for the patient.

For peer review only - http://bmjopen.bmj.com/site/about/guidelines.xhtml

All interviews will be transcribed verbatim, stored and organised using NVivo software (QSR International Pty Ltd, Doncaster, Victoria, Australia). Interview and visual data from GP and patient interviews will be subjected to thematic analysis, with a particular orientation to exploring clinical and patient perspectives on the barriers and facilitators to reducing antihypertensives.

Study 2: Assessment of trial recruitment and data collection procedures
The aim of the second qualitative study will be to explore how information is presented within recruitment appointments and how this might impact on consent to participate, with a view to ensuring robust trial procedures using an iterative process. This will be achieved by audio-recording (with consent) up to 75 consultations between GPs or research assistants and eligible patients.

Thematic analysis will be undertaken on a sample of around 15-20 consultations comprising patients who did, or did not consent to participate, to consider (a) terminology used, (b) presentation of the de-prescribing approach and (c) presentation of randomisation. This will inform on-going trial procedures and future implementation.

Economic sub-study
This work will adapt a previous decision-analytic model examining the long-term costs and benefits from blood pressure lowering treatment[32] to include potential harms of treatment. The model will be adjusted for the effects of blood pressure lowering on cardiovascular disease risk, costs and quality-adjusted-life years (QALYs) to match the older population involved in this work. Costs of the therapies prescribed, side-effects and acute and long term costs of cardiovascular events will be obtained within the trial and from the literature. Quality of life on each treatment strategy will be obtained from the trial data using EQ-5D-5L, and previous studies will inform utility values for cardiovascular disease health states and the impact of side effects. The model will determine the cost per additional QALY gained of the medication reduction intervention versus usual care and analyses will be conducted from a health and social services perspective. The model will be run with a lifetime perspective, with costs and benefits discounted at a rate of 3.5%. A value of information analysis will assess whether a further trial would be appropriate to reduce decision uncertainty, and identify those parameters where more precise estimates would be most valuable and should therefore be chosen as outcomes for such a trial.

Ethics and dissemination
This research involves older participants, some of whom may be considered vulnerable. Great care will be taken to ensure all potential participants have the trial clearly explained, and are given sufficient time to decide whether to give informed consent. This will include provision of simplified, participant information sheets with large fonts, video infographics to explain the study and extended GP consultation periods for explaining the study and taking informed consent. The protocol, informed consent form, participant information sheet and all other participant facing material have been approved by the Research Ethics Committee (South Central - Oxford A; ref 16/SC/0628), Medicines and Healthcare products Regulatory Agency (ref 21584/0371/001-0001), host institution(s) and Health Research Authority.

All research outputs from this work will be published in peer-reviewed journals, presented at scientific conferences and lay and social media (e.g. Twitter, blogs). 'Patient friendly' study summary documents and infographics will be made available to all participants at the end of the trial via the study website.

For peer review only - http://bmjopen.bmj.com/site/about/guidelines.xhtml

Discussion

Current guidelines in the UK suggest that doctors should ensure that patients are fully informed of the benefits and risks of their prescribed medications and where appropriate, discuss the potential for medication withdrawal in frail individuals with multi-morbidity. [33] This is difficult given consultation time constraints and fear that de-prescribing might result in harm.[34] This is compounded by conflicting and inconclusive evidence about the benefits and harms of treatment, and a lack of evidence about what will happen if these treatments are reduced.

The ECSTATIC trial enrolled 1,067 younger participants aged 40-70 years, taking antihypertensives for primary prevention of cardiovascular disease.[18] The trial demonstrated that only 27% of participants were able to maintain medication reduction throughout follow-up and at 3 months, systolic blood pressure was on average 6 mmHg higher in the de-prescribing group. At 2 year follow-up, the risk of uncontrolled blood pressure was significantly higher in those patients attempting to de-prescribe. Unlike the present study, the medication reduction algorithm used did not encourage reintroduction of therapy if blood pressure was persistently raised.

The DANTE study[16] examined the effect of complete antihypertensive medication discontinuation in 385 patients over the age of 75 years and with mild cognitive deficits. After 16 weeks of follow-up, they observed a 7/3 mmHg increase in blood pressure but no difference in overall cognition compound score or quality of life between groups. A study by Van der Wardt and colleagues[19] examined the feasibility trial reducing antihypertensives in patients with dementia, but was only able to recruit 9 participants for the withdrawal programme (1% recruitment rate) and a larger trial was deemed unfeasible. Similarly, the OPTIMED trial[35] demonstrated in 95 participants that a broader de-prescribing approach is achievable in patients living in nursing homes, but was unable to examine the effect on clinical outcomes due to recruitment issues resulting on only 38% of the planned sample size being enrolled.

The OPTiMISE trial will target frail individuals with polypharmacy and co-morbidity, and aim to establish whether a strategy of antihypertensive medication reduction is safe and acceptable to older patients. The findings of this trial will support better patient-centred management plans for the prevention of cardiovascular disease in older individuals and inform future de-prescribing trials in primary care.

For peer review only - http://bmjopen.bmj.com/site/about/guidelines.xhtml

Acknowledgements

The authors acknowledge the support of the Primary Care Clinical Trials Unit, staff from the NIHR CRNs including Thames Valley and South Midlands, Cambridge, Southampton, West Midlands (Central and South) and West of England, and Lucy Curtin for administrative support. Margaret Ogden and Anita Higham serve as patient representatives for the trial steering committee. Additional members of the trial steering committee are Prof Tom Robinson (chair), Prof Rod Taylor and Dr Peter Bower. Members of the data monitoring committee are Prof John Gladman (chair), Prof Una Martin and Dr Martyn Lewis. The sponsor and funder had no role in the study design, writing of the paper; or the decision to submit this protocol for publication, which was made jointly by the authors who have all approved the final manuscript. Finally, this work would not be possible without the support of the participating practices and participants.

Funding

This work receives joint funding from the National Institute for Health Research (NIHR) Oxford Collaboration for Leadership in Applied Health Research and Care (CLAHRC) at Oxford Health NHS Foundation Trust (ref: P2-501) and the NIHR School for Primary Care Research (SPCR; ref 335). JS and RJMcM have been funded by an NIHR Professorship (NIHR-RP-R2-12-015). FDRH acknowledges part support from the NIHR SPCR, the NIHR CLAHRC Oxford, and the NIHR Oxford Biomedical Research Centre (BRC). CH receives support from the NIHR SPCR and NIHR Oxford BRC. The views and opinions expressed are those of the authors and do not necessarily reflect those of the NHS, NIHR, or the Department of Health.

Competing interests

The authors declare no conflicts of interest.

Author's contributions

JS conceived, designed and secured funding for the study with JBu, ML, JBe, GF, CH, FDRH, SJ, PL, JM, EO, RP, MW, LMY and RJMcM. JS wrote the first draft. AN and LMY provided the sample size calculations and statistical analysis section. JBu provided the qualitative section. SJ provided the health economic section. All authors reviewed and edited the manuscript. ET is the trial manager. JS and RJMcM are co-chief investigators and will act as guarantors for this work.

Data sharing

Data sharing requests will be considered by the corresponding author.

For peer review only

References

1. Office for National Statistics. Mid-year populations estimates: Aging, fastest increase in the 'oldest old'. http://www.ons.gov.uk, 2010.

2. Barnett K, Mercer SW, Norbury M, et al. Epidemiology of multimorbidity and implications for health care, research, and medical education: a cross-sectional study. *Lancet* 2012;380(9836):37-43. doi: 10.1016/s0140-6736(12)60240-2 [published Online First: 2012/05/15]

3. Violan C, Foguet-Boreu Q, Roso-Llorach A, et al. Burden of multimorbidity, socioeconomic status and use of health services across stages of life in urban areas: a cross-sectional study. *BMC public health* 2014;14:530. doi: 10.1186/1471-2458-14-530 [published Online First: 2014/06/03]

4. Sheppard JP, Singh S, Fletcher K, et al. Impact of age and sex on primary preventive treatment for cardiovascular disease in the West Midlands, UK: cross sectional study. *BMJ (Clinical research ed)* 2012;345:e4535. doi: 10.1136/bmj.e4535 [published Online First: 2012/07/14]

5. Beckett NS, Peters R, Fletcher AE, et al. Treatment of hypertension in patients 80 years of age or older. *The New England journal of medicine* 2008;358(18):1887-98. doi: 10.1056/NEJMoa0801369 [published Online First: 2008/04/02]

6. A Randomized Trial of Intensive versus Standard Blood-Pressure Control. *The New England journal of medicine* 2015 doi: 10.1056/NEJMoa1511939 [published Online First: 2015/11/10]

7. McKee M, Britton A, Black N, et al. Interpreting the evidence: choosing between randomised and non-randomised studies. *BMJ (Clinical research ed)* 1999;319(7205):312-5.

8. van Deudekom FJ, Postmus I, van der Ham DJ, et al. External validity of randomized controlled trials in older adults, a systematic review. *PloS one* 2017;12(3):e0174053. doi: 10.1371/journal.pone.0174053 [published Online First: 2017/03/28]

9. Bejan-Angoulvant T, Saadatian-Elahi M, Wright JM, et al. Treatment of hypertension in patients 80 years and older: the lower the better? A meta-analysis of randomized controlled trials. *Journal of hypertension* 2010;28(7):1366-72. doi: 10.1097/HJH.0b013e328339f9c5 [published Online First: 2010/06/25]

10. Benetos A, Labat C, Rossignol P, et al. Treatment With Multiple Blood Pressure Medications, Achieved Blood Pressure, and Mortality in Older Nursing Home Residents: The PARTAGE Study. *JAMA internal medicine* 2015 doi: 10.1001/jamainternmed.2014.8012 [published Online First: 2015/02/17]

11. Tinetti ME, Han L, Lee DS, et al. Antihypertensive medications and serious fall injuries in a nationally representative sample of older adults. *JAMA internal medicine* 2014;174(4):588-95. doi: 10.1001/jamainternmed.2013.14764 [published Online First: 2014/02/26]

12. Fried TR, Tinetti ME, Towle V, et al. Effects of benefits and harms on older persons' willingness to take medication for primary cardiovascular prevention. *Archives of internal medicine* 2011;171(10):923-8. doi: 10.1001/archinternmed.2011.32 [published Online First: 2011/03/02]

13. Schuling J, Gebben H, Veehof LJ, et al. Deprescribing medication in very elderly patients with multimorbidity: the view of Dutch GPs. A qualitative study. *BMC family practice* 2012;13:56. doi: 10.1186/1471-2296-13-56 [published Online First: 2012/06/16]

14. Scott IA, Anderson K, Freeman CR, et al. First do no harm: a real need to deprescribe in older patients. *The Medical journal of Australia* 2014;201(7):390-2. [published Online First: 2014/10/09]

For peer review only - http://bmjopen.bmj.com/site/about/guidelines.xhtml

15. Iyer S, Naganathan V, McLachlan AJ, et al. Medication withdrawal trials in people aged 65 years and older: a systematic review. *Drugs & aging* 2008;25(12):1021-31. doi: 10.2165/0002512-200825120-00004 [published Online First: 2008/11/22]

16. Moonen JE, Foster-Dingley JC, de Ruijter W, et al. Effect of Discontinuation of Antihypertensive Treatment in Elderly People on Cognitive Functioning--the DANTE Study Leiden: A Randomized Clinical Trial. *JAMA internal medicine* 2015;175(10):1622-30. doi: 10.1001/jamainternmed.2015.4103 [published Online First: 2015/08/25]

17. van der Wardt V, Harrison JK, Welsh T, et al. Withdrawal of antihypertensive medication: a systematic review. *Journal of hypertension* 2017;35(9):1742-49. doi: 10.1097/hjh.0000000000001405 [published Online First: 2017/05/10]

18. Luymes CH, Poortvliet RKE, van Geloven N, et al. Deprescribing preventive cardiovascular medication in patients with predicted low cardiovascular disease risk in general practice - the ECSTATIC study: a cluster randomised non-inferiority trial. *BMC medicine* 2018;16(1):5. doi: 10.1186/s12916-017-0988-0 [published Online First: 2018/01/13]

19. van der Wardt V, Burton JK, Conroy S, et al. Withdrawal of antihypertensive therapy in people with dementia: feasibility study. *Pilot and feasibility studies* 2018;4:29. doi: 10.1186/s40814-017-0221-0 [published Online First: 2018/01/18]

20. Clegg A, Bates C, Young J, et al. Development and validation of an electronic frailty index using routine primary care electronic health record data. *Age and ageing* 2016;45(3):353-60. doi: 10.1093/ageing/afw039 [published Online First: 2016/03/06]

21. National Clinical Guideline C. National Institute for Health and Clinical Excellence Guidance CG127. Hypertension: The Clinical Management of Primary Hypertension in Adults: Update of Clinical Guidelines 18 and 34. London: Royal College of Physicians (UK) 2011.

22. Mattu GS, Heran BS, Wright JM. Overall accuracy of the BpTRU--an automated electronic blood pressure device. *Blood pressure monitoring* 2004;9(1):47-52. [published Online First: 2004/03/17]

23. Lahrmann H, Cortelli P, Hilz M, et al. EFNS guidelines on the diagnosis and management of orthostatic hypotension. *European journal of neurology* 2006;13(9):930-6. doi: 10.1111/j.1468-1331.2006.01512.x [published Online First: 2006/08/26]

24. Herdman M, Gudex C, Lloyd A, et al. Development and preliminary testing of the new five-level version of EQ-5D (EQ-5D-5L). *Quality of life research : an international journal of quality of life aspects of treatment, care and rehabilitation* 2011;20(10):1727-36. doi: 10.1007/s11136-011-9903-x [published Online First: 2011/04/12]

25. Gallagher P, Ryan C, Byrne S, et al. STOPP (Screening Tool of Older Person's Prescriptions) and START (Screening Tool to Alert doctors to Right Treatment). Consensus validation. *International journal of clinical pharmacology and therapeutics* 2008;46(2):72-83. [published Online First: 2008/01/26]

26. McManus RJ, Mant J, Bray EP, et al. Telemonitoring and self-management in the control of hypertension (TASMINH2): a randomised controlled trial. *Lancet* 2010;376(9736):163-72. doi: 10.1016/s0140-6736(10)60964-6 [published Online First: 2010/07/14]

27. McManus RJ, Mant J, Haque MS, et al. Effect of self-monitoring and medication self-titration on systolic blood pressure in hypertensive patients at high risk of cardiovascular disease: the TASMIN-SR randomized clinical trial. *Jama* 2014;312(8):799-808. doi: 10.1001/jama.2014.10057 [published Online First: 2014/08/27]

For peer review only

For peer review only - http://bmjopen.bmj.com/site/about/guidelines.xhtml

28. Cuspidi C, Meani S, Lonati L, et al. Prevalence of home blood pressure measurement among selected hypertensive patients: results of a multicenter survey from six hospital outpatient hypertension clinics in Italy. *Blood pressure* 2005;14(4):251-6. doi: 10.1080/08037050500210765 [published Online First: 2005/08/30]

29. Tucker KL, Sheppard JP, Stevens R, et al. Self-monitoring of blood pressure in hypertension: A systematic review and individual patient data meta-analysis. *PLoS medicine* 2017;14(9):e1002389. doi: 10.1371/journal.pmed.1002389 [published Online First: 2017/09/20]

30. Walker E, Nowacki AS. Understanding equivalence and noninferiority testing. *Journal of general internal medicine* 2011;26(2):192-6. doi: 10.1007/s11606-010-1513-8 [published Online First: 2010/09/22]

31. Nathan A, Goodyer L, Lovejoy A, et al. 'Brown bag' medication reviews as a means of optimizing patients' use of medication and of identifying potential clinical problems. *Family practice* 1999;16(3):278-82. [published Online First: 1999/08/10]

32. Kaambwa B, Bryan S, Jowett S, et al. Telemonitoring and self-management in the control of hypertension (TASMINH2): a cost-effectiveness analysis. *European journal of preventive cardiology* 2014;21(12):1517-30. doi: 10.1177/2047487313501886 [published Online First: 2013/08/31]

33. National Guideline Centre. National Institute for Health and Care Excellence: Clinical Guidelines. Multimorbidity: Assessment, Prioritisation and Management of Care for People with Commonly Occurring Multimorbidity [NICE Guideline 56]. London: National Institute for Health and Care Excellence (UK)

Copyright (c) National Institute for Health and Care Excellence, 2016. 2016.

34. Palagyi A, Keay L, Harper J, et al. Barricades and brickwalls--a qualitative study exploring perceptions of medication use and deprescribing in long-term care. *BMC geriatrics* 2016;16:15. doi: 10.1186/s12877-016-0181-x [published Online First: 2016/01/16]

35. Potter K, Flicker L, Page A, et al. Deprescribing in Frail Older People: A Randomised Controlled Trial. *PloS one* 2016;11(3):e0149984. doi: 10.1371/journal.pone.0149984 [published Online First: 2016/03/05]

36. Sulter G, Steen C, De Keyser J. Use of the Barthel index and modified Rankin scale in acute stroke trials. *Stroke; a journal of cerebral circulation* 1999;30(8):1538-41. [published Online First: 1999/08/06]

37. Morley JE, Malmstrom TK, Miller DK. A simple frailty questionnaire (FRAIL) predicts outcomes in middle aged African Americans. *The journal of nutrition, health & aging* 2012;16(7):601-8. [published Online First: 2012/07/28]

38. Rockwood K, Song X, MacKnight C, et al. A global clinical measure of fitness and frailty in elderly people. *CMAJ : Canadian Medical Association journal = journal de l'Association medicale canadienne* 2005;173(5):489-95. doi: 10.1503/cmaj.050051 [published Online First: 2005/09/01]

39. Nasreddine ZS, Phillips NA, Bedirian V, et al. The Montreal Cognitive Assessment, MoCA: a brief screening tool for mild cognitive impairment. *Journal of the American Geriatrics Society* 2005;53(4):695-9. doi: 10.1111/j.1532-5415.2005.53221.x [published Online First: 2005/04/09]

40. Horne R, Weinman J. Patients' beliefs about prescribed medicines and their role in adherence to treatment in chronic physical illness. *Journal of psychosomatic research* 1999;47(6):555-67. [published Online First: 2000/02/08]

For peer review only - http://bmjopen.bmj.com/site/about/guidelines.xhtml

Tables

Table 1. Trial inclusion and exclusion criteria

**Inclusion criteria**
• Participant is willing and able to give informed consent for participation in the trial.
• Male or Female, aged 80 years or above.
• Clinic systolic blood pressure less than 150 mmHg (according to screening measurement at baseline – clinic blood pressure defined as the mean of the 2nd and 3rd readings taken at 1 minute intervals).
• Prescribed two or more antihypertensive medications to lower blood pressure for at least 12 months prior to trial entry. Antihypertensive medications defined as any ACE inhibitor, angiotensin II receptor blocker, calcium channel blocker, thiazide and thiazide-like diuretic, potassium-sparing diuretic, alpha-blocker, beta-blocker, vasodilator antihypertensives, centrally acting antihypertensives, direct renin inhibitors, adrenergic neurone blocking drugs or loop diuretics.
• Stable dose of antihypertensive medications for at least four weeks prior to trial entry.
• In the Investigator’s opinion, could potentially benefit from medication reduction due to existing polypharmacy, co-morbidity, non-adherence or dislike of medicines and/or frailty
• In the Investigator’s opinion, is able and willing to comply with all trial requirements.
**Exclusion criteria**
• A participant has heart failure due to LVSD and is on only ACE inhibitors/ARBs and/or beta-blockers and/or spironolactone (removing any of which would be contraindicated).
• A participant has heart failure but has not had an echocardiogram since its onset (might have undiagnosed LVSD and a compelling need for ACE inhibitors/ARB and Beta-blockers).
• Investigator deems that there is a compelling indication for antihypertensive medication continuation.
• Any other significant disease or disorder which, in the opinion of the Investigator, may either put the participants at risk because of participation in the trial, or may influence the result of the trial, or the participant’s ability to participate in the trial (e.g. terminal illness, house bound and unable to attend baseline and follow up clinics).
• Suffered a myocardial infarction or stroke within the past 12 months.
• Blood pressure being managed outside of primary care.
• Unable to provide consent due to incapacity.
• A participant with secondary hypertension or previous accelerated or malignant hypertension.
• Participants who have participated in another research trial involving antihypertensive medication in the past 4 weeks.

LVSD=Left ventricular systolic dysfunction; ACE inhibitor=Angiotensin Converting Enzyme inhibitor; ARB=Angiotensin II receptor blocker

For peer review only - http://bmjopen.bmj.com/site/about/guidelines.xhtml

Table 2. Variables and schedule of data collection

No.	Variable	Data source		Schedule	
		Medical notes	Measured/collected at clinic	Baseline	Follow-up
1	Age		✓	✓	
2	Sex		✓	✓	
3	Ethnicity		✓	✓	
4	Marital status		✓	✓	
5	Education		✓	✓	
6	Duration of hypertension	✓		✓	
7	Past medical history	✓		✓	
8	Alcohol consumption		✓	✓	✓
9	Smoking		✓	✓	✓
10	Height		✓	✓	✓
11	Weight		✓	✓	✓
12	Clinic blood pressure (sitting and standing)		✓	✓	✓
13	Cholesterol (total and HDL)	✓		✓	
14	estimated Glomerular Filtration Rate (eGFR)	✓		✓	
15	Prescribed or over the counter medications (all medications)*	✓	✓	✓	✓
16	Quality of life (according to EQ-5D-5L)[24]		✓	✓	✓
17	Functional independence (defined by modified Rankin Scale)[36]		✓	✓	
18	Frailty (according to the FRAIL scale)[37]		✓	✓	✓
19	Frailty (according to the frailty index and electronic frailty index)[20 38]	✓	✓	✓	✓
20	Cognitive function (defined by the Montreal Cognitive Assessment [MoCA])[39]		✓	✓	
21	Adherence to medication (according to the Medication Adherence Rating Scale (MARS) Questionnaire)[40]		✓	✓	✓
22	Adherence to medication reduction		✓		✓
23	ICD-10 coded Cardiovascular events and mortality during the trial	✓			✓
24	Recording of potential side effects to medication		✓	✓	✓
25	Recording of adverse events	✓	✓		✓

HDL = High density lipoprotein; ICD = International Statistical Classification of Diseases and Related Health Problems

*Drug substance/name, formulation, dose, frequency, start date and adherence over past 12 months (according to clinical system)

For peer review only

For peer review only - http://bmjopen.bmj.com/site/about/guidelines.xhtml

Figure legends

Figure 1. Trial flow diagram

*Monitoring of blood pressure at home will be encouraged but those not willing or able will still be included in the trial. All participants will be asked to attend a safety monitoring visit with their GP/nurse four weeks after baseline.
GP = General practitioner; BP = Blood pressure; HDL = High density lipoprotein; ICD = International Statistical Classification of Diseases and Related Health Problems; CVD = Cardiovascular disease; eGFR = estimated Glomerular Filtration Rate (eGFR); MARS = Medication Adherence Rating Scale; MOCA = Montreal Cognitive Assessment

Figure 2. Medication reduction algorithm

STOPP criteria[25]
Withdraw the one of the following medications if any of the ensuing contraindications are identified:

- Thiazide diuretic with a history of gout (may exacerbate gout).
- Beta-blocker in combination with verapamil (risk of symptomatic heart block).
- Non-cardioselective beta-blocker with chronic obstructive pulmonary disease (risk of bronchospasm).
- Calcium channel blockers with chronic constipation (may exacerbate constipation).
- Use of diltiazem or verapamil with NYHA Class III or IV heart failure (may worsen heart failure).

Figure 3. Post medication reduction monitoring flow chart

Note: The full effects of most oral antihypertensives can last for up to 4-6 weeks. Frequent monitoring in the initial 4 weeks after drug withdrawal is thus not required unless BP levels are extreme or there are other clinical concerns (see above). Where systolic/diastolic BP values fall into different categories, consider the higher value. BP should be taken as the averaged second and third measurements using a validated monitor. Standard clinical care/monitoring should align with NICE recommendations.[21]

For peer review only - http://bmjopen.bmj.com/site/about/guidelines.xhtml

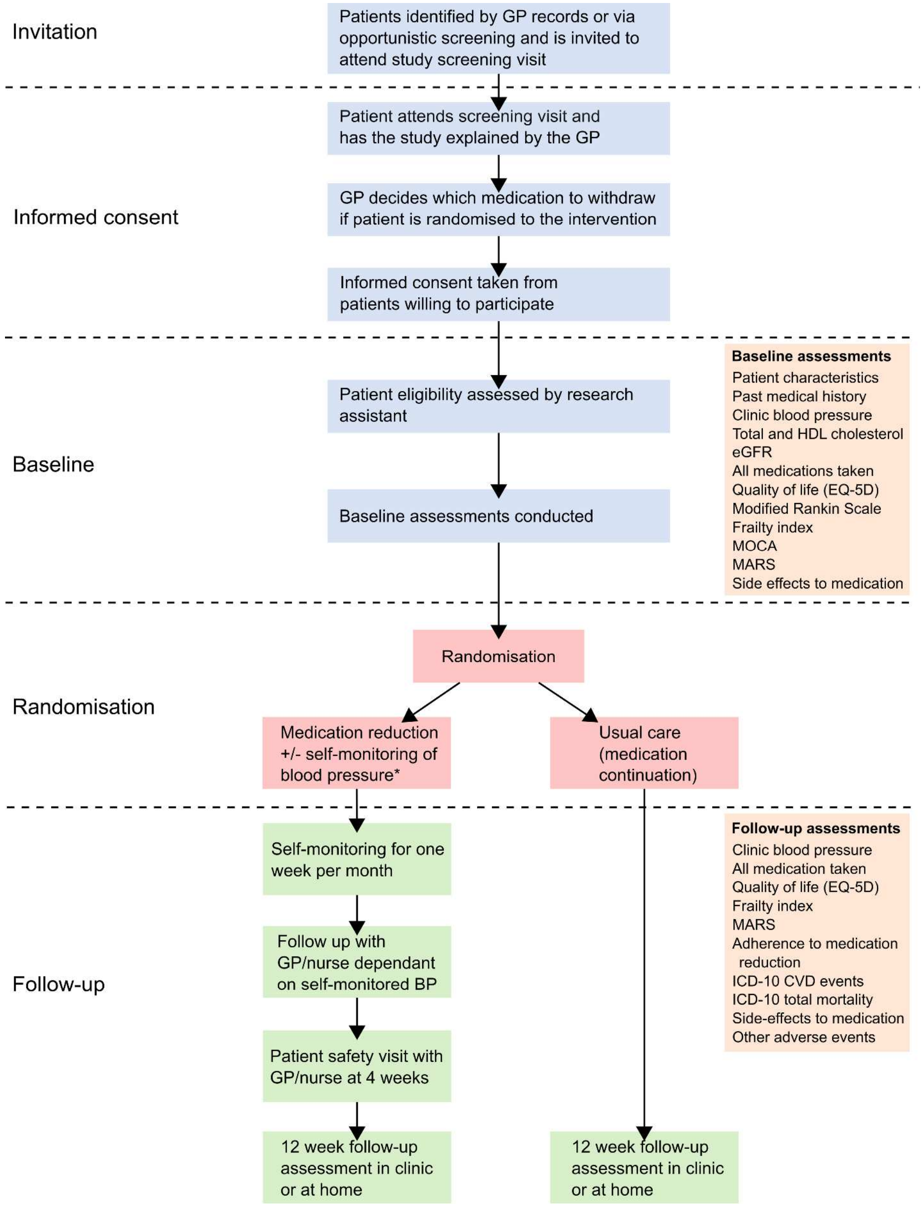
Invitation
Patients identified by GP records or via opportunistic screening and is invited to attend study screening visit
Informed consent
Patient attends screening visit and has the study explained by the GP
GP decides which medication to withdraw if patient is randomised to the intervention
Informed consent taken from patients willing to participate
Baseline
Patient eligibility assessed by research assistant
Baseline assessments conducted
Baseline assessments
Patient characteristics
Past medical history
Clinic blood pressure
Total and HDL cholesterol
eGFR
All medications taken
Quality of life (EQ-5D)
Modified Rankin Scale
Frailty index
MOCA
MARS
Side effects to medication
Randomisation
Randomisation
Medication reduction +/- self-monitoring of blood pressure*
Usual care (medication continuation)
Follow-up
Self-monitoring for one week per month
Follow up with GP/nurse dependant on self-monitored BP
Patient safety visit with GP/nurse at 4 weeks
12 week follow-up assessment in clinic or at home
12 week follow-up assessment in clinic or at home
Follow-up assessments
Clinic blood pressure
All medication taken
Quality of life (EQ-5D)
Frailty index
MARS
Adherence to medication reduction
ICD-10 CVD events
ICD-10 total mortality
Side-effects to medication
Other adverse events

Figure 1

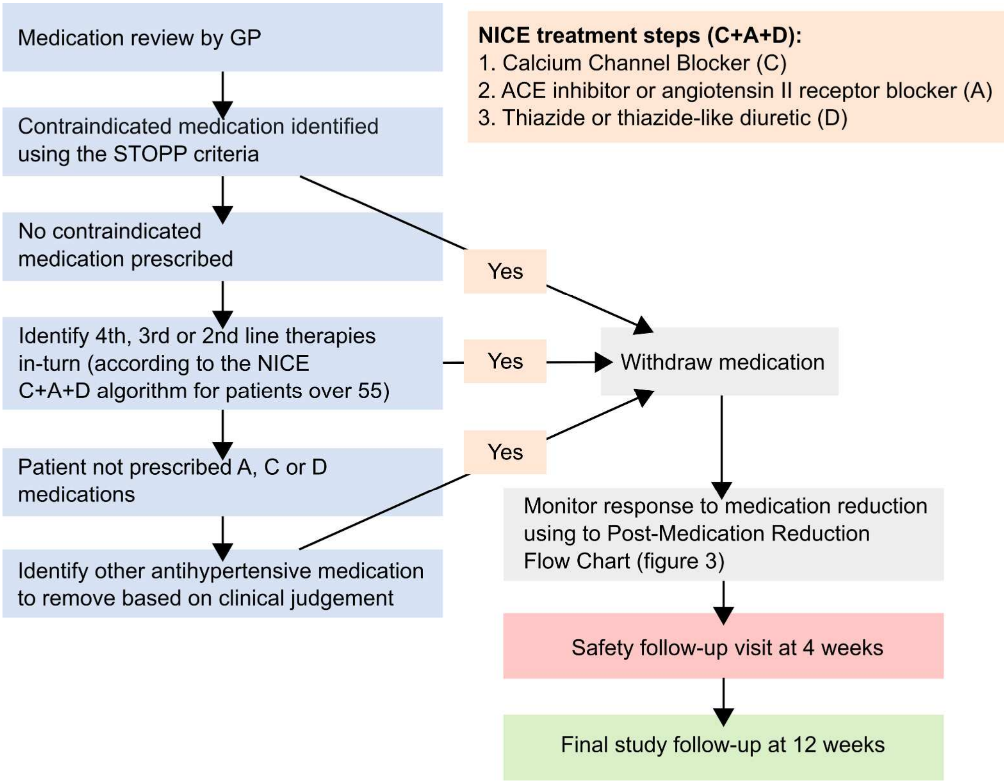

Figure 2

ew only

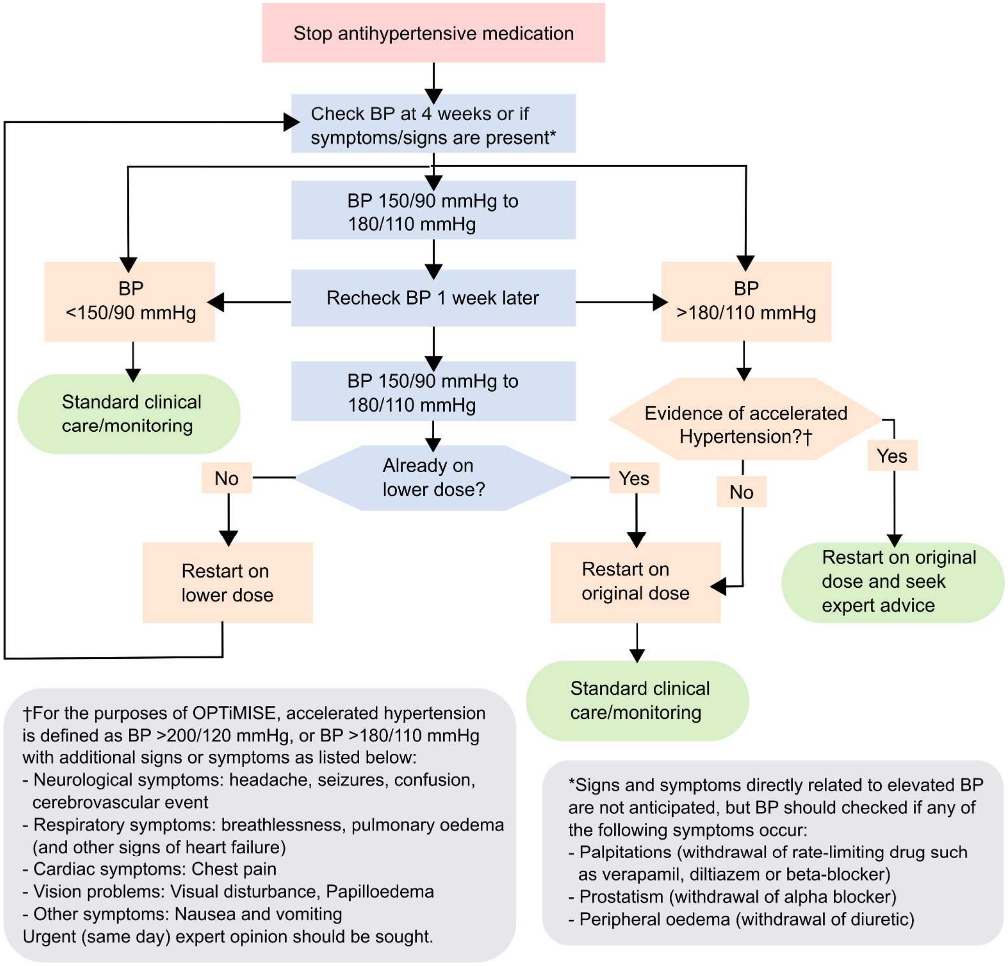
Stop antihypertensive medication
Check BP at 4 weeks or if symptoms/signs are present*
BP 150/90 mmHg to 180/110 mmHg
Recheck BP 1 week later
BP <150/90 mmHg
BP >180/110 mmHg
Standard clinical care/monitoring
BP 150/90 mmHg to 180/110 mmHg
Evidence of accelerated Hypertension?†
Already on lower dose?
No
Yes
No
Yes
Restart on lower dose
Restart on original dose
Restart on original dose and seek expert advice
Standard clinical care/monitoring
†For the purposes of OPTiMISE, accelerated hypertension is defined as BP >200/120 mmHg, or BP >180/110 mmHg with additional signs or symptoms as listed below:
- Neurological symptoms: headache, seizures, confusion, cerebrovascular event
- Respiratory symptoms: breathlessness, pulmonary oedema (and other signs of heart failure)
- Cardiac symptoms: Chest pain
- Vision problems: Visual disturbance, Papilloedema
- Other symptoms: Nausea and vomiting
Urgent (same day) expert opinion should be sought.
*Signs and symptoms directly related to elevated BP are not anticipated, but BP should checked if any of the following symptoms occur:
- Palpitations (withdrawal of rate-limiting drug such as verapamil, diltiazem or beta-blocker)
- Prostatism (withdrawal of alpha blocker)
- Peripheral oedema (withdrawal of diuretic)

Figure 3

SPIRIT 2013 Checklist: Recommended items to address in a clinical trial protocol and related documents*

Section/item	Item No	Description	Page in protocol
**Administrative information**			
Title	1	Descriptive title identifying the study design, population, interventions, and, if applicable, trial acronym	1
Trial registration	2a	Trial identifier and registry name. If not yet registered, name of intended registry	2
	2b	All items from the World Health Organization Trial Registration Data Set	n/a
Protocol version	3	Date and version identifier	n/a
Funding	4	Sources and types of financial, material, and other support	12
Roles and responsibilities	5a	Names, affiliations, and roles of protocol contributors	1, 12
	5b	Name and contact information for the trial sponsor	1
	5c	Role of study sponsor and funders, if any, in study design; collection, management, analysis, and interpretation of data; writing of the report; and the decision to submit the report for publication, including whether they will have ultimate authority over any of these activities	12
	5d	Composition, roles, and responsibilities of the coordinating centre, steering committee, endpoint adjudication committee, data management team, and other individuals or groups overseeing the trial, if applicable (see Item 21a for data monitoring committee)	12
**Introduction**			
Background and rationale	6a	Description of research question and justification for undertaking the trial, including summary of relevant studies (published and unpublished) examining benefits and harms for each intervention	3
	6b	Explanation for choice of comparators	3
Objectives	7	Specific objectives or hypotheses	3-4
Trial design	8	Description of trial design including type of trial (eg, parallel group, crossover, factorial, single group), allocation ratio, and framework (eg, superiority, equivalence, noninferiority, exploratory)	4
**Methods: Participants, interventions, and outcomes**			
Study setting	9	Description of study settings (eg, community clinic, academic hospital) and list of countries where data will be collected. Reference to where list of study sites can be obtained	4
Eligibility criteria	10	Inclusion and exclusion criteria for participants. If applicable, eligibility criteria for study centres and individuals who will	4, table 1

For peer review only - http://bmjopen.bmj.com/site/about/guidelines.xhtml

		perform the interventions (eg, surgeons, psychotherapists)	
Interventions	11a	Interventions for each group with sufficient detail to allow replication, including how and when they will be administered	5
	11b	Criteria for discontinuing or modifying allocated interventions for a given trial participant (eg, drug dose change in response to harms, participant request, or improving/worsening disease)	Figure 3
	11c	Strategies to improve adherence to intervention protocols, and any procedures for monitoring adherence (eg, drug tablet return, laboratory tests)	6-7
	11d	Relevant concomitant care and interventions that are permitted or prohibited during the trial	7
Outcomes	12	Primary, secondary, and other outcomes, including the specific measurement variable (eg, systolic blood pressure), analysis metric (eg, change from baseline, final value, time to event), method of aggregation (eg, median, proportion), and time point for each outcome. Explanation of the clinical relevance of chosen efficacy and harm outcomes is strongly recommended	4-5
Participant timeline	13	Time schedule of enrolment, interventions (including any run-ins and washouts), assessments, and visits for participants. A schematic diagram is highly recommended (see Figure)	Figure 1
Sample size	14	Estimated number of participants needed to achieve study objectives and how it was determined, including clinical and statistical assumptions supporting any sample size calculations	8
Recruitment	15	Strategies for achieving adequate participant enrolment to reach target sample size	5
**Methods: Assignment of interventions (for controlled trials)**			
Allocation:			
Sequence generation	16a	Method of generating the allocation sequence (eg, computer-generated random numbers), and list of any factors for stratification. To reduce predictability of a random sequence, details of any planned restriction (eg, blocking) should be provided in a separate document that is unavailable to those who enrol participants or assign interventions	6
Allocation concealment mechanism	16b	Mechanism of implementing the allocation sequence (eg, central telephone; sequentially numbered, opaque, sealed envelopes), describing any steps to conceal the sequence until interventions are assigned	6
Implementation	16c	Who will generate the allocation sequence, who will enrol participants, and who will assign participants to interventions	6
Blinding (masking)	17a	Who will be blinded after assignment to interventions (eg, trial participants, care providers, outcome assessors, data analysts), and how	6

For peer review only

	17b	If blinded, circumstances under which unblinding is permissible, and procedure for revealing a participant's allocated intervention during the trial	6
**Methods: Data collection, management, and analysis**			
Data collection methods	18a	Plans for assessment and collection of outcome, baseline, and other trial data, including any related processes to promote data quality (eg, duplicate measurements, training of assessors) and a description of study instruments (eg, questionnaires, laboratory tests) along with their reliability and validity, if known. Reference to where data collection forms can be found, if not in the protocol	5-7
	18b	Plans to promote participant retention and complete follow-up, including list of any outcome data to be collected for participants who discontinue or deviate from intervention protocols	7
Data management	19	Plans for data entry, coding, security, and storage, including any related processes to promote data quality (eg, double data entry; range checks for data values). Reference to where details of data management procedures can be found, if not in the protocol	6
Statistical methods	20a	Statistical methods for analysing primary and secondary outcomes. Reference to where other details of the statistical analysis plan can be found, if not in the protocol	8
	20b	Methods for any additional analyses (eg, subgroup and adjusted analyses)	8
	20c	Definition of analysis population relating to protocol non-adherence (eg, as randomised analysis), and any statistical methods to handle missing data (eg, multiple imputation)	8
**Methods: Monitoring**			
Data monitoring	21a	Composition of data monitoring committee (DMC); summary of its role and reporting structure; statement of whether it is independent from the sponsor and competing interests; and reference to where further details about its charter can be found, if not in the protocol. Alternatively, an explanation of why a DMC is not needed	9
	21b	Description of any interim analyses and stopping guidelines, including who will have access to these interim results and make the final decision to terminate the trial	n/a
Harms	22	Plans for collecting, assessing, reporting, and managing solicited and spontaneously reported adverse events and other unintended effects of trial interventions or trial conduct	9
Auditing	23	Frequency and procedures for auditing trial conduct, if any, and whether the process will be independent from investigators and the sponsor	9
**Ethics and dissemination**			
Research ethics approval	24	Plans for seeking research ethics committee/institutional review board (REC/IRB) approval	10

For peer review only

Protocol amendments	25	Plans for communicating important protocol modifications (eg, changes to eligibility criteria, outcomes, analyses) to relevant parties (eg, investigators, REC/IRBs, trial participants, trial registries, journals, regulators)	n/a
Consent or assent	26a	Who will obtain informed consent or assent from potential trial participants or authorised surrogates, and how (see Item 32)	5
	26b	Additional consent provisions for collection and use of participant data and biological specimens in ancillary studies, if applicable	n/a
Confidentiality	27	How personal information about potential and enrolled participants will be collected, shared, and maintained in order to protect confidentiality before, during, and after the trial	n/a
Declaration of interests	28	Financial and other competing interests for principal investigators for the overall trial and each study site	12
Access to data	29	Statement of who will have access to the final trial dataset, and disclosure of contractual agreements that limit such access for investigators	12
Ancillary and post-trial care	30	Provisions, if any, for ancillary and post-trial care, and for compensation to those who suffer harm from trial participation	n/a
Dissemination policy	31a	Plans for investigators and sponsor to communicate trial results to participants, healthcare professionals, the public, and other relevant groups (eg, via publication, reporting in results databases, or other data sharing arrangements), including any publication restrictions	10
	31b	Authorship eligibility guidelines and any intended use of professional writers	n/a
	31c	Plans, if any, for granting public access to the full protocol, participant-level dataset, and statistical code	n/a
**Appendices**			
Informed consent materials	32	Model consent form and other related documentation given to participants and authorised surrogates	n/a
Biological specimens	33	Plans for collection, laboratory evaluation, and storage of biological specimens for genetic or molecular analysis in the current trial and for future use in ancillary studies, if applicable	n/a

*It is strongly recommended that this checklist be read in conjunction with the SPIRIT 2013 Explanation & Elaboration for important clarification on the items. Amendments to the protocol should be tracked and dated. The SPIRIT checklist is copyrighted by the SPIRIT Group under the Creative Commons "Attribution-NonCommercial-NoDerivs 3.0 Unported" license.

For peer review only

BMJ Open

OPtimising Treatment for MIld Systolic hypertension in the Elderly (OPTiMISE): protocol for a randomised controlled non-inferiority trial

Journal:	*BMJ Open*
Manuscript ID	bmjopen-2018-022930.R1
Article Type:	Protocol
Date Submitted by the Author:	14-Jun-2018
Complete List of Authors:	Sheppard, James; University of Oxford, Nuffield Department of Primary Care Health Sciences<br>Burt, Jenni; University of Cambridge, Cambridge Centre for Health Services Research<br>`Lown, Mark; University of Southampton<br>Temple, Eleanor; University of Oxford, Nuffield Department of Primary Care Health Sciences<br>Benson, John; University of Cambridge, GP and Primary Care Research Unit<br>Ford, Gary; Oxford University Hospitals NHS Foundation Trust, Oxford Academic Health Science Network<br>Heneghan, Carl; Oxford University, Primary Health Care<br>Hobbs, Richard; University of Oxford, Nuffield Department of Primary Care Health Sciences<br>Jowett, Sue; University of Birmingham, Health Economics Unit<br>Little, Paul; University of Southampton, Primary Care and Population Science;<br>Mant, Jonathan; University of Cambridge, General Practice and Primary Care Research Unit<br>Mollison, Jill; University of Oxford, Nuffield Department of Primary Care Health Sciences<br>Nickless, Alecia; University of Oxford, Nuffield Department of Primary Care Health Sciences<br>Ogburn, Emma; University of Oxford,<br>Payne, Rupert; University of Bristol, Centre for Academic Primary Care<br>Williams, Marney; Patient and public involvement representative<br>Yu, Ly-Mee; University of Oxford, Department of Primary Care Health Sciences<br>McManus, Richard; University of Oxford, Dept of Primary Care Health Sciences
**Primary Subject Heading**:	General practice / Family practice
Secondary Subject Heading:	Cardiovascular medicine, Geriatric medicine, Health services research
Keywords:	Multi-morbidity, Cardiovascular disease, Frailty, Antihypertensive, De-prescribing

Note: The following files were submitted by the author for peer review, but cannot be converted to PDF. You must view these files (e.g. movies) online.
OPTiMISE video inforgraphic V1.1 14.03.17.mp4

SCHOLARONE™ Manuscripts

For peer review only

OPtimising Treatment for MIld Systolic hypertension in the Elderly (OPTiMISE): protocol for a randomised controlled non-inferiority trial

James P Sheppard,[1] Jenni Burt,[2] Mark Lown,[3] Eleanor Temple,[1] John Benson,[4] Gary A Ford,[1] Carl Heneghan,[1] FD Richard Hobbs,[1] Sue Jowett,[5] Paul Little,[3] Jonathan Mant,[4] Jill Mollison,[1] Alecia Nickless,[1] Emma Ogburn,[1] Rupert Payne,[6] Marney Williams,[7] Ly-Mee Yu,[1] and Richard J McManus[1]

[1]Nuffield Department of Primary Care Health Sciences, University of Oxford, Oxford, UK
[2]The Healthcare Improvement Studies Institute, University of Cambridge, Cambridge, UK
[3]Primary Care Research Group, University of Southampton, Southampton, UK
[4]Primary Care Unit, Department of Public Health and Primary Care, University of Cambridge, Cambridge, UK
[5]Institute of Applied Health Research, University of Birmingham, Birmingham, UK
[6]Centre for Academic Primary Care, University of Bristol, Bristol, UK
[7]Patient and public involvement representative, London, UK

Corresponding author: James P Sheppard
Email: james.sheppard@phc.ox.ac.uk
Telephone: +44 1865 617192
Address: Nuffield Department of Primary Care Health Sciences, Radcliffe Primary Care Building, Radcliffe Observatory Quarter, University of Oxford, Oxford, OX2 6GG, UK

Trial Sponsor: University of Oxford
Contact name: Ms Heather House
Address: Clinical Trials and Research Governance, Joint Research Office, Block 60, Churchill Hospital, University of Oxford, Oxford, OX3 7LE
Email: ctrg@admin.ox.ac.uk

Word count: 4,208 (excluding title page, abstract, references, tables and figures)
Number of tables: 2
Number of figures: 3

For peer review only

For peer review only - http://bmjopen.bmj.com/site/about/guidelines.xhtml

Abstract

Introduction: Recent evidence suggests that larger blood pressure reductions and multiple antihypertensive drugs may be harmful in older people, particularly frail individuals with polypharmacy and multi-morbidity. However, there is a lack of evidence to support de-prescribing of antihypertensives, which limits the practice of medication reduction in routine clinical care. The aim of this trial is to examine whether antihypertensive medication reduction is possible in older patients without significant changes in blood pressure control at follow-up.

Methods and analysis: This trial will use a Primary Care based, open label, randomised controlled trial design. A total of 540 participants will be recruited, aged ≥80 years, with systolic blood pressure <150 mmHg and receiving ≥2 antihypertensive medications. Participants will have no compelling indication for medication continuation and will be considered to potentially benefit from medication reduction due to existing polypharmacy, co-morbidity and frailty. Following a baseline appointment, individuals will be randomised to a strategy of medication reduction (intervention) with optional self-monitoring or usual care (control). Those in the intervention group will have one antihypertensive medication stopped. The primary outcome will be to determine if a reduction in medication can achieve a proportion of participants with clinically safe blood pressure levels at 12 week follow-up (defined as a systolic blood pressure <150mmHg) which is non-inferior (within 10%) to that achieved by the usual care group. Qualitative interviews will be used to understand the barriers and facilitators to medication reduction. The study will use economic modelling to predict the long term effects of any observed changes in blood pressure and quality-of-life.

Ethics and dissemination: The protocol and written information has been approved by a Research Ethics Committee, medicines regulatory authority (MHRA), and national and local health research authorities. All research outputs will be published in peer-reviewed journals and presented at national and international conferences.

Trial registration: EudraCT 2016-004236-38

Word count: 299 (max 300)

Keywords: Multi-morbidity, cardiovascular disease, frailty, antihypertensive, de-prescribing

For peer review only - http://bmjopen.bmj.com/site/about/guidelines.xhtml

Strengths and limitations of this study

- This will be the first UK randomised controlled trial to compare a strategy of antihypertensive medication reduction to usual care in primary care.
- The pragmatic trial design, with broad inclusion criteria, will make findings of the study externally valid in routine clinical practice.
- Allowing the attending GP to choose the medication to be reduced will maximise external validity of the trial results but precludes the possibility of blinding the participants and investigators.
- The trial will be powered to detect a non-inferior difference in blood pressure control at follow-up, but not necessarily secondary outcomes such as differences in rates of cardiovascular disease, adverse events and quality of life.

For peer review only

Introduction

The general population is ageing[1] and, consequently, the number of people living with age-related chronic conditions is increasing.[2] Hypertension is the number one co-morbid condition in older people with multiple chronic conditions[3] and 52% of those aged ≥80 years are prescribed two or more antihypertensive drugs (equivalent to approximately 1.25 million people in the UK).[4] Blood pressure lowering has been shown to be effective at preventing stroke and cardiovascular disease in healthy individuals aged ≥80 years with stage 2 hypertension (systolic blood pressure of >160mmHg) and high risk stage 1 hypertension.[5,6] However, as with many trials,[7,8] these studies included healthier populations with lower polypharmacy and multi-morbidity than might be expected in the general elderly population. In addition, there is evidence to suggest that larger blood pressure reductions and multiple antihypertensive prescriptions may be harmful in older people.[9,10] Evidence from observational studies also suggests that higher intensity blood pressure treatment is associated with increased risk of falls in older people,[11] although this is also disputed.[5]

Some patients consider the increased risk of falls and other adverse events to be as important as the risk of myocardial infarction or stroke, particularly those taking medications for primary prevention of cardiovascular disease.[12] Thus, decisions over blood pressure lowering in the elderly, particularly the frail elderly, require the weighing of harms and quality of life. However, clinicians can often struggle to stop prescribing medication due to a perceived lack of evidence, fear of the reaction of other prescribers, fear of precipitating events such as stroke or angina and concern that patients will feel their care is being cut.[13,14]

There is limited evidence from randomised trials examining the safety of antihypertensive medication reduction or withdrawal.[15-19] The HYVET trial[5] enrolled some patients on antihypertensive treatment who were then randomised to placebo (effectively complete medication withdrawal) and the ANBP2 trial investigators followed up participants who withdrew medication during the trial run-in period but who were not randomised into the trial. They found younger patients with lower baseline blood pressure and fewer drug prescriptions were more likely to sustain medication withdrawal at 12 month follow-up.[20,21] However, there are few trials comparing a specified strategy of antihypertensive medication reduction with usual care in terms of effects on blood pressure control and quality of life.[17] In addition, there are no previous economic modelling studies of a strategy of medication reduction in the elderly.

The aim of this work will be to examine whether antihypertensive medication reduction in patients with controlled systolic hypertension (<u><</u>150mmHg) who are being prescribed two or more antihypertensives is possible without significant changes in blood pressure control at follow-up.

Methods

Aims and outcomes

The aim of this study is to determine whether antihypertensive medications can be safely reduced without systolic blood pressure increasing beyond what is clinical acceptable at follow-up.

The primary outcome is the proportion of participants with clinically acceptable levels at 12 week follow-up (defined as a systolic blood pressure <150mmHg) Secondary outcomes will examine:

- The proportion of participants in the intervention arm who maintain medication reduction through to follow-up (*i.e.* are *not* restarted on therapy).
- The difference in quality of life (according to EQ-5D-5L) between groups at 12 week follow-up.
- The difference in frailty (according to the frailty index)[22] between the two groups at 12 week follow-up.

For peer review only

- The difference in the change in mean clinic systolic blood pressure (from baseline) between the two groups at 12 week follow-up.
- The difference in reported side effects to medication between the two groups at 12 week follow-up (including coughs, dizziness, syncope, and ankle swelling).
- The difference in routinely reported serious adverse events between the two groups at 12 week follow-up (hospitalisation due to falls, myocardial infarction, stroke or all-cause mortality).

Design

This trial will use a Primary Care based, open label, randomised controlled, two-parallel groups, non-inferiority trial design, recruiting 540 participants with controlled blood pressure (systolic <150 mmHg) on two or more antihypertensive treatments. Participants will be randomised to a strategy of medication reduction (intervention) or usual care (control) and followed-up for 12 weeks (see figure 1). Embedded qualitative and economic analyses will examine barriers and facilitators to medication reduction and the cost effectiveness of the approach.

Trial participants

Patients eligible for the trial will be aged ≥80 years, with systolic blood pressure <150mmHg (current UK guideline recommendation)[23] receiving ≥2 antihypertensive medications. They will have no compelling indication for medication continuation and in the opinion of the attending GP, may potentially benefit from medication reduction due to existing polypharmacy, co-morbidity and/or frailty (table 1).

Participants will be identified and recruited from general practices via the UK Clinical Research Network (CRN). Potentially eligible patients will be identified by trained practice staff searching practice-based electronic disease registers using a standardised strategy. GPs will be asked to check the search results and remove people whom they believe to be unsuitable to participate in the study. Remaining potentially eligible patients will be sent letters of invitation from their GP and those expressing an interest in the trial will be asked to attend a screening and baseline appointment. Patients not responding to the first invitation will receive one reminder letter (up to four weeks after the first letter). Other potentially eligible patients may also be approached opportunistically by a member of the clinical care team. Those who do not wish to take part will be asked to fill in a short questionnaire detailing their reasons.

Baseline visit

Eligible patients will have informed consent taken by the GP. During the consent appointment, the GP will show a two-minute study video infographic (see supplementary material) and go through the participant information sheet explaining the exact nature of the trial. Having had a chance to ask questions, those individuals willing to participate will give written informed consent by means of a participant dated signature and dated signature of the GP who presented and obtained the informed consent.

Some participants will be invited to have their interview audio-recorded for qualitative analysis during their study visits. Those who are interested will be asked to sign a response slip prior to meeting the GP. Consent to audio recordings will not have a bearing on an individual’s care or eligibility for the main trial.

Those giving informed consent will be screened using the criteria in table 1 and undergo baseline measurements and randomisation by a member of the research team via participant questionnaires and a detailed notes review (table 2). Blood pressure will be measured in a standardised fashion using the clinically validated[24] BpTRU blood pressure monitor which automatically records six blood pressure

For peer review only - http://bmjopen.bmj.com/site/about/guidelines.xhtml

measurements at one minute intervals. Blood pressure readings will be taken in the left arm (where appropriate) after participants have been seated for at least five minutes of rest, using an appropriate sized cuff. The mean of the 2nd and 3rd readings will be used to define the primary outcome. To test for orthostatic hypotension, two further readings will be taken in the standing position after one and three minutes.[25] Only the research facilitator/nurse will be present during the blood pressure measurements. Orthostatic hypotension will be defined as a ≥20mmHg drop in systolic blood pressure within three minutes of standing.

All data will be collected via an electronic case report form (eCRF) linked to the study database. Participants will be given the option to enter responses to questionnaires themselves or with assistance from the research team. Where questionnaires are not validated for use on a tablet computer,[26] or where individuals are not comfortable using one, paper copies will be made available for completion.

Randomisation

Consenting participants will be individually randomised (1:1 allocation ratio) to one of two study arms using a fully validated web-based system (Sortition®) with manual telephone back up. Participants will not be randomised until after consent has been taken and baseline assessments have been completed. A computer generated non-deterministic algorithm, minimising on practice and baseline systolic blood pressure will be used to ensure these covariates are balanced between the two intervention arms.

The study will use an open label design, so patients and practitioners will not be blinded to the intervention or study endpoints. Therefore, codebreaking will not be necessary. The statistical analysis will be performed blind to patient allocation.

Intervention group

Participating GPs will review each participant's antihypertensive medication regimen prior to the baseline appointment, and decide which medication should be removed if they are randomised to the intervention arm of the trial. The choice of medication to be withdrawn will be at the discretion of the GP, but their decision will be informed by an individual's co-morbidities and existing guidelines, where appropriate (figure 2). Specifically, participating GPs will be encouraged to identify previously unrecognised contraindications to medication, defined by the STOPP criteria.[27] In the absence of these, or a strong clinical rationale for continuing despite a STOPP criteria being met, GPs will be recommended to reduce antihypertensive medications in reverse of the NICE C+A+D algorithm for older patients (figure 2).[23] All participants in the trial will remain on at least one antihypertensive.

Once a medication has been removed, GPs or other appropriate, delegated healthcare professionals will closely monitor the participant's response to medication reduction: they will be given advice about what and when to monitor (figure 3), but this schedule will be flexible. All participants will be expected to return for at least one routine safety follow-up visit, and further visits may be required if blood pressure is raised (≥150 mmHg), or adverse events occur. Where blood pressure is persistently raised, GPs will be expected to re-adjust medication (dose or type), rendering the likelihood of a serious adverse event occurring as a result of the intervention very low.

Self-monitoring

All participants randomised to the medication reduction arm of the trial will be given the option to self-monitor their blood pressure at home. Those accepting will be trained using protocols developed in the

For peer review only

For peer review only - http://bmjopen.bmj.com/site/about/guidelines.xhtml

previous TASMIN trials[28 29] and will be given simple and clear instructions to contact their GP if their blood pressure rises above what is considered clinically safe (e.g. home systolic blood pressure >145mmHg on all readings taken in a week). Participants will be advised to self-monitor (or have a carer monitor) at least 4 times per week in the last week of each month of follow-up (weeks 4, 8 and 12), although they can monitor more frequently if they wish. Differential use of self-monitoring in the intervention group, or indeed in the control group (many patients now self-monitor routinely)[30] is not expected to impact on the study results, since there is good evidence that self-monitoring only affects blood pressure levels if used in combination with a co-intervention.[31] All other clinical care will continue as usual.

Control group

Those allocated to the control arm of the study will continue usual clinical care (i.e. they will continue to take antihypertensive medications as prescribed and will not self-monitor unless already doing so). No other medication changes will be mandated and participating GPs will be asked to manage all other care according to usual clinical practice.

Follow-up visits

Participants will attend one research follow-up clinic, 12 weeks (±2 weeks) after baseline and those in the intervention will attend one additional safety visit after four weeks (±2 weeks) (figure 1). A period of four weeks is expected to be sufficiently long enough to assess the impact of antihypertensive medication reduction, since these drugs usually take approximately four weeks to 'wash out' of a patient's system. Earlier safety visits are not recommended since they could provide false reassurance that blood pressure is within safe limits if the withdrawn drug has not washed out of the participant's system.

The follow-up assessments will include standardised blood pressure measurement (for assessment of the primary outcome), questionnaire assessments and adherence to the trial medication regime, side effects and adverse events (table 2). Where possible, all participants will be flagged for mortality and hospital admissions using NHS patient tracking services, permitting long-term follow-up for up to 5 years after the trial has finished.

Each participant has the right to withdraw from the trial at any time. We will ask all participants to attend a follow-up visit as far as is practicable, regardless of whether medication is re-introduced to participants in the intervention group, or a participant in the control group has medication withdrawn. Unless a participant withdraws consent, vital status will be assessed even where an individual has been lost to follow-up (for instance moved away). If given, the reason for withdrawal will be recorded in the eCRF.

Internal feasibility study

A two-stage internal feasibility study will be conducted to examine methods of patient invitation and rates of recruitment, before proceeding with the main trial. The first feasibility phase will last for a minimum of three months and aim to recruit approximately 25 participants from a minimum of 3-5 practices. The aim will be to establish whether or not anyone will be willing to participate in the study.

The second feasibility phase will focus on recruitment rates for the main trial and whether the intended sample size is likely to be met during the recruitment period. This phase will have a recruitment target of 75 participants from ten practices over 6 months, giving a total sample for the feasibility study of 100 participants. A recruitment rate of 15% of invitations sent is expected. The following actions will be considered to address varying rates of recruitment at the end of the feasibility phases:

For peer review only - http://bmjopen.bmj.com/site/about/guidelines.xhtml

- If ≥100 participants are recruited – trial will proceed as planned
- If 75-99 participants are recruited – recruitment materials/method will be re-examined and edited where necessary following discussions with stakeholders and patient and public involvement representatives.
- If 50-74 participants are recruited – the allocation of resources and recruitment criteria will be re-examined using information gathered from concurrent qualitative work.
- If <50 participants are recruited – the Trial Steering Committee (TSC) will decide, in discussion with the Data Monitoring and Ethics Committee (DMEC) and the funders, whether the trial should be stopped due to futility.

Sample size calculation

Assuming that 100% of participants in the usual care group, and 96% of those in the medication reduction group have controlled systolic blood pressure levels (<150mmHg) at follow-up, approximately 540 participants will be required to detect a non-inferior difference in systolic blood pressure control between groups. Calculations assume a 10% non-inferiority margin, 90% power, 2.5% 1-sided level of significance, 10% loss to follow-up and a 10% dilution effect due to cross-over between arms. There is no existing precedent for an appropriate margin of non-inferiority in this type of trial, but 10% was deemed useful to inform future doctor-patient discussions about medication reduction: if the non-inferiority margin is met, it will suggest that for every ten patients who have their medication reduced, at least nine will still have controlled blood pressure at 12 week follow-up.

Statistical analysis

A detailed statistical analysis plan will be agreed prior to the end of the trial. The primary and secondary analyses will be by intention to treat (ITT), unless stated otherwise. The primary analysis will be a non-inferiority analysis by means of the "two one-sided test" (TOST) procedure,[32] whereby the 95% confidence interval for the relative risk of participants with systolic blood pressure at 12 weeks below 150 mmHg between the medication reduction group and the usual care group is calculated. This will be obtained by means of a generalised linear mixed effects model with GP surgery included as a random effect and baseline blood pressure as a fixed effect. If the lower limit of the confidence interval is more than 0.9 (equal to a risk difference of 10%) then the research hypothesis that medication reduction will be non-inferior in terms of blood pressure control to usual care will be accepted. As a secondary analysis of the primary outcome, a per-protocol (PP) analysis will be performed, since ITT can be anticonservative for a non-inferiority hypothesis.[32] Participants who received the medication reduction intervention in the PP analysis will be defined as a participant in the medication reduction arm who maintained their medication reduction throughout the 12 week follow-up period.

Secondary analyses will examine the proportion of participants in the medication reduction arm who maintained their medication reduction throughout the 12 week follow-up period. Secondary outcomes will be analysed by means of linear mixed effects models, adjusting for the baseline level of the outcome and baseline systolic blood pressure and including practice as a random effect: systolic blood pressure, EQ-5D-5L and the Frailty index/frail scale. The difference in the rate of side effects and adverse events between the medication reduction and usual care arms will be analysed by means of a logistic mixed effects model adjusting for baseline systolic blood pressure and including practice as a random effect.

For peer review only - http://bmjopen.bmj.com/site/about/guidelines.xhtml

Exploratory subgroup analyses of blood pressure control, change in blood pressure and maintenance of medication reduction will be conducted by different levels of baseline frailty, functional independence, cognitive function, number of medications prescribed at baseline and number of co-morbidities at baseline.

Patient and public involvement

This protocol was developed through discussions with older patients and carers and members of an AgeUK day centre. MW is a stroke survivor with experience as a volunteer on the elderly ward of Charing Cross Hospital. She was consulted on the suitability and design of the trial and is a member of the trial management group. Methods of patient approach, including the design of the video infographic, patient information sheet and consent form were all reviewed by patient representatives prior to formal approval. The TSC includes two independent patient representatives responsible for overseeing the conduct of the trial.

Safety reporting

Adverse events that are observed by an investigator or reported by the participant will be recorded on the AE log at any time during the study but AEs will be specifically asked about at the 12 week follow up. Serious adverse events (SAEs) will be reported to the coordinating centre within 24 hours of discovery or notification of the event. All SAE reports will be reviewed by the DMEC chair on a monthly basis, and by the full DMEC at meetings held every 6 months. The DMEC will include a geriatrician, statistician and consultant clinical pharmacologist. They will be responsible for safeguarding trial participants, monitoring emerging trial data including identifying any trends, such as increases in unexpected events, and take appropriate action where necessary.

All adverse events labelled possibly, probably or definitely related will be considered as related to the intervention. Since there are no sections of the Summary of medicinal Product Characteristics, or previous clinical studies which detail expected adverse events as a result of medication withdrawal, all SAEs at least possibly related, and not as a result of re-introduction of withdrawn medication, will be considered unexpected and reported as SUSARs. Fatal and life-threatening SUSARs will be reported by the chief investigator to the relevant Competent Authority and Research Ethics Committee no later than 7 calendar days after the Sponsor or delegate is first aware of the reaction. All other SUSARs will be reported within 15 calendar days.

Qualitative sub-studies

Study 1: interviews with doctors and patient

Face-to-face interviews with GPs and patients will be conducted to generate understanding about the barriers and facilitators to antihypertensive medication reduction. Informed consent will be sought from approximately 15 GPs to provide a broad range of opinion from varying practice sizes (small to large) and settings (rural to urban). Participating GPs will also be asked to identify up to 15 patients for interview, based on the same inclusion criteria as those applied to participants in the main trial.

Interviews with GPs will use a chart-stimulated recall approach to explore the factors which influence their treatment choices in older hypertensive patients. Anonymised electronic health records will be used to focus discussions about how GPs would feel about reducing antihypertensive medications. Interviews with patients will use ‘brown bag’ medication review techniques[33] to create a complete record of medication held, with a commentary on usage from the participants’ perspective. Diagrammatic elicitation techniques will be used to complete a relational map outlining participants’ circumstances and how these relate to the

For peer review only - http://bmjopen.bmj.com/site/about/guidelines.xhtml

medications taken. These sketches will be used as the basis for a discussion on the implications of withdrawing antihypertensive medications, and what this "gap" might mean for the patient.

All interviews will be transcribed verbatim, stored and organised using NVivo software (QSR International Pty Ltd, Doncaster, Victoria, Australia). Interview and visual data from GP and patient interviews will be subjected to thematic analysis, with a particular orientation to exploring clinical and patient perspectives on the barriers and facilitators to reducing antihypertensives.

Study 2: Assessment of trial recruitment and data collection procedures

The aim of the second qualitative study will be to explore how information is presented within recruitment appointments and how this might impact on consent to participate, with a view to ensuring robust trial procedures using an iterative process. This will be achieved by audio-recording (with consent) up to 75 consultations between GPs or research assistants and eligible patients.

Thematic analysis will be undertaken on a sample of around 15-20 consultations comprising patients who did, or did not consent to participate, to consider (a) terminology used, (b) presentation of the de-prescribing approach and (c) presentation of randomisation. This will inform on-going trial procedures and future implementation.

Economic sub-study

This work will adapt a previous decision-analytic model examining the long-term costs and benefits from blood pressure lowering treatment[34] to include potential harms of treatment. The model will be adjusted for the effects of blood pressure lowering on cardiovascular disease risk, costs and quality-adjusted-life years (QALYs) to match the older population involved in this work. Costs of the therapies prescribed, side-effects and acute and long term costs of cardiovascular events will be obtained within the trial and from the literature. Quality of life on each treatment strategy will be obtained from the trial data using EQ-5D-5L, and previous studies will inform utility values for cardiovascular disease health states and the impact of side effects. The model will determine the cost per additional QALY gained of the medication reduction intervention versus usual care and analyses will be conducted from a health and social services perspective. The model will be run with a lifetime perspective, with costs and benefits discounted at a rate of 3.5%. A value of information analysis will assess whether a further trial would be appropriate to reduce decision uncertainty, and identify those parameters where more precise estimates would be most valuable and should therefore be chosen as outcomes for such a trial.

Ethics and dissemination

This research involves older participants, some of whom may be considered vulnerable. Great care will be taken to ensure all potential participants have the trial clearly explained, and are given sufficient time to decide whether to give informed consent. This will include provision of simplified, participant information sheets with large fonts, video infographics to explain the study and extended GP consultation periods for explaining the study and taking informed consent. The protocol, informed consent form, participant information sheet and all other participant facing material have been approved by the Research Ethics Committee (South Central - Oxford A; ref 16/SC/0628), Medicines and Healthcare products Regulatory Agency (ref 21584/0371/001-0001), host institution(s) and Health Research Authority. The study sponsor reviewed and ensured all indemnity and insurance requirements for the trial were in place prior to the start of recruitment.

For peer review only - http://bmjopen.bmj.com/site/about/guidelines.xhtml

All research outputs from this work will be published in peer-reviewed journals, presented at scientific conferences and lay and social media (e.g. Twitter, blogs). 'Patient friendly' study summary documents and infographics will be made available to all participants at the end of the trial via the study website.

Current trial status

The trial commenced recruitment on 10th April 2017 and is estimated to continue recruitment until September 2018.

Discussion

Current guidelines in the UK suggest that doctors should ensure that patients are fully informed of the benefits and risks of their prescribed medications and where appropriate, discuss the potential for medication withdrawal in frail individuals with multi-morbidity. [35] This is difficult given consultation time constraints and fear that de-prescribing might result in harm.[36] This is compounded by conflicting and inconclusive evidence about the benefits and harms of treatment, and a lack of evidence about what will happen if these treatments are reduced.

The ECSTATIC trial enrolled 1,067 younger participants aged 40-70 years, taking antihypertensives for primary prevention of cardiovascular disease.[18] The trial demonstrated that only 27% of participants were able to maintain medication reduction throughout follow-up and at 3 months, systolic blood pressure was on average 6 mmHg higher in the de-prescribing group. At 2 year follow-up, the risk of uncontrolled blood pressure was significantly higher in those patients attempting to de-prescribe. Unlike the present study, the medication reduction algorithm used did not encourage reintroduction of therapy if blood pressure was persistently raised.

The DANTE study[16] examined the effect of complete antihypertensive medication discontinuation in 385 patients over the age of 75 years and with mild cognitive deficits. After 16 weeks of follow-up, they observed a 7/3 mmHg increase in blood pressure but no difference in overall cognition compound score or quality of life between groups. A study by Van der Wardt and colleagues[19] examined the feasibility trial reducing antihypertensives in patients with dementia, but was only able to recruit 9 participants for the withdrawal programme (1% recruitment rate) and a larger trial was deemed unfeasible. Similarly, the OPTIMED trial[37] demonstrated in 95 participants that a broader de-prescribing approach is achievable in patients living in nursing homes, but was unable to examine the effect on clinical outcomes due to recruitment issues resulting on only 38% of the planned sample size being enrolled.

The OPTiMISE trial will target frail individuals with polypharmacy and co-morbidity, and aim to establish whether a strategy of antihypertensive medication reduction is safe and acceptable to older patients. The findings of this trial will support better patient-centred management plans for the prevention of cardiovascular disease in older individuals and inform future de-prescribing trials in primary care.

For peer review only - http://bmjopen.bmj.com/site/about/guidelines.xhtml

Acknowledgements

The authors acknowledge the support of the Primary Care Clinical Trials Unit, staff from the NIHR CRNs including Thames Valley and South Midlands, Cambridge, Southampton, West Midlands (Central and South) and West of England, and Lucy Curtin for administrative support. Margaret Ogden and Anita Higham serve as patient representatives for the trial steering committee. Additional members of the trial steering committee are Prof Tom Robinson (chair), Prof Rod Taylor and Dr Peter Bower. Members of the data monitoring committee are Prof John Gladman (chair), Prof Una Martin and Dr Martyn Lewis. The sponsor and funder had no role in the study design, writing of the paper; or the decision to submit this protocol for publication, which was made jointly by the authors who have all approved the final manuscript. Finally, this work would not be possible without the support of the participating practices and participants.

Funding

This work receives joint funding from the National Institute for Health Research (NIHR) Oxford Collaboration for Leadership in Applied Health Research and Care (CLAHRC) at Oxford Health NHS Foundation Trust (ref: P2-501) and the NIHR School for Primary Care Research (SPCR; ref 335). JS and RJMcM have been funded by an NIHR Professorship (NIHR-RP-R2-12-015). FDRH acknowledges part support from the NIHR SPCR, the NIHR CLAHRC Oxford, and the NIHR Oxford Biomedical Research Centre (BRC). CH receives support from the NIHR SPCR and NIHR Oxford BRC. The views and opinions expressed are those of the authors and do not necessarily reflect those of the NHS, NIHR, or the Department of Health.

Competing interests

The authors declare no conflicts of interest.

Author's contributions

JS conceived, designed and secured funding for the study with JBu, ML, JBe, GF, CH, FDRH, SJ, PL, JM, EO, RP, MW, LMY and RJMcM. JS wrote the first draft. AN and LMY provided the sample size calculations and statistical analysis section. JBu provided the qualitative section. SJ provided the health economic section. All authors reviewed and edited the manuscript. ET is the trial manager. JS and RJMcM are co-chief investigators and will act as guarantors for this work.

Data sharing

Data sharing requests will be considered by the corresponding author.

For peer review only

For peer review only - http://bmjopen.bmj.com/site/about/guidelines.xhtml

References

1. Office for National Statistics. Mid-year populations estimates: Aging, fastest increase in the 'oldest old'. http://www.ons.gov.uk, 2010.

2. Barnett K, Mercer SW, Norbury M, et al. Epidemiology of multimorbidity and implications for health care, research, and medical education: a cross-sectional study. *Lancet* 2012;380(9836):37-43. doi: 10.1016/s0140-6736(12)60240-2 [published Online First: 2012/05/15]

3. Violan C, Foguet-Boreu Q, Roso-Llorach A, et al. Burden of multimorbidity, socioeconomic status and use of health services across stages of life in urban areas: a cross-sectional study. *BMC public health* 2014;14:530. doi: 10.1186/1471-2458-14-530 [published Online First: 2014/06/03]

4. Sheppard JP, Singh S, Fletcher K, et al. Impact of age and sex on primary preventive treatment for cardiovascular disease in the West Midlands, UK: cross sectional study. *BMJ (Clinical research ed)* 2012;345:e4535. doi: 10.1136/bmj.e4535 [published Online First: 2012/07/14]

5. Beckett NS, Peters R, Fletcher AE, et al. Treatment of hypertension in patients 80 years of age or older. *The New England journal of medicine* 2008;358(18):1887-98. doi: 10.1056/NEJMoa0801369 [published Online First: 2008/04/02]

6. A Randomized Trial of Intensive versus Standard Blood-Pressure Control. *The New England journal of medicine* 2015 doi: 10.1056/NEJMoa1511939 [published Online First: 2015/11/10]

7. McKee M, Britton A, Black N, et al. Interpreting the evidence: choosing between randomised and non-randomised studies. *BMJ (Clinical research ed)* 1999;319(7205):312-5.

8. van Deudekom FJ, Postmus I, van der Ham DJ, et al. External validity of randomized controlled trials in older adults, a systematic review. *PloS one* 2017;12(3):e0174053. doi: 10.1371/journal.pone.0174053 [published Online First: 2017/03/28]

9. Bejan-Angoulvant T, Saadatian-Elahi M, Wright JM, et al. Treatment of hypertension in patients 80 years and older: the lower the better? A meta-analysis of randomized controlled trials. *Journal of hypertension* 2010;28(7):1366-72. doi: 10.1097/HJH.0b013e328339f9c5 [published Online First: 2010/06/25]

10. Benetos A, Labat C, Rossignol P, et al. Treatment With Multiple Blood Pressure Medications, Achieved Blood Pressure, and Mortality in Older Nursing Home Residents: The PARTAGE Study. *JAMA internal medicine* 2015 doi: 10.1001/jamainternmed.2014.8012 [published Online First: 2015/02/17]

11. Tinetti ME, Han L, Lee DS, et al. Antihypertensive medications and serious fall injuries in a nationally representative sample of older adults. *JAMA internal medicine* 2014;174(4):588-95. doi: 10.1001/jamainternmed.2013.14764 [published Online First: 2014/02/26]

12. Fried TR, Tinetti ME, Towle V, et al. Effects of benefits and harms on older persons' willingness to take medication for primary cardiovascular prevention. *Archives of internal medicine* 2011;171(10):923-8. doi: 10.1001/archinternmed.2011.32 [published Online First: 2011/03/02]

13. Schuling J, Gebben H, Veehof LJ, et al. Deprescribing medication in very elderly patients with multimorbidity: the view of Dutch GPs. A qualitative study. *BMC family practice* 2012;13:56. doi: 10.1186/1471-2296-13-56 [published Online First: 2012/06/16]

14. Scott IA, Anderson K, Freeman CR, et al. First do no harm: a real need to deprescribe in older patients. *The Medical journal of Australia* 2014;201(7):390-2. [published Online First: 2014/10/09]

For peer review only - http://bmjopen.bmj.com/site/about/guidelines.xhtml

15. Iyer S, Naganathan V, McLachlan AJ, et al. Medication withdrawal trials in people aged 65 years and older: a systematic review. *Drugs & aging* 2008;25(12):1021-31. doi: 10.2165/0002512-200825120-00004 [published Online First: 2008/11/22]

16. Moonen JE, Foster-Dingley JC, de Ruijter W, et al. Effect of Discontinuation of Antihypertensive Treatment in Elderly People on Cognitive Functioning--the DANTE Study Leiden: A Randomized Clinical Trial. *JAMA internal medicine* 2015;175(10):1622-30. doi: 10.1001/jamainternmed.2015.4103 [published Online First: 2015/08/25]

17. van der Wardt V, Harrison JK, Welsh T, et al. Withdrawal of antihypertensive medication: a systematic review. *Journal of hypertension* 2017;35(9):1742-49. doi: 10.1097/hjh.0000000000001405 [published Online First: 2017/05/10]

18. Luymes CH, Poortvliet RKE, van Geloven N, et al. Deprescribing preventive cardiovascular medication in patients with predicted low cardiovascular disease risk in general practice - the ECSTATIC study: a cluster randomised non-inferiority trial. *BMC medicine* 2018;16(1):5. doi: 10.1186/s12916-017-0988-0 [published Online First: 2018/01/13]

19. van der Wardt V, Burton JK, Conroy S, et al. Withdrawal of antihypertensive therapy in people with dementia: feasibility study. *Pilot and feasibility studies* 2018;4:29. doi: 10.1186/s40814-017-0221-0 [published Online First: 2018/01/18]

20. Nelson MR, Reid CM, Krum H, et al. Predictors of normotension on withdrawal of antihypertensive drugs in elderly patients: prospective study in second Australian national blood pressure study cohort. *BMJ (Clinical research ed)* 2002;325(7368):815. [published Online First: 2002/10/12]

21. Nelson MR, Reid CM, Krum H, et al. Short-term predictors of maintenance of normotension after withdrawal of antihypertensive drugs in the second Australian National Blood Pressure Study (ANBP2). *American journal of hypertension* 2003;16(1):39-45. [published Online First: 2003/01/09]

22. Clegg A, Bates C, Young J, et al. Development and validation of an electronic frailty index using routine primary care electronic health record data. *Age and ageing* 2016;45(3):353-60. doi: 10.1093/ageing/afw039 [published Online First: 2016/03/06]

23. National Clinical Guideline C. National Institute for Health and Clinical Excellence Guidance CG127. Hypertension: The Clinical Management of Primary Hypertension in Adults: Update of Clinical Guidelines 18 and 34. London: Royal College of Physicians (UK) 2011.

24. Mattu GS, Heran BS, Wright JM. Overall accuracy of the BpTRU--an automated electronic blood pressure device. *Blood pressure monitoring* 2004;9(1):47-52. [published Online First: 2004/03/17]

25. Lahrmann H, Cortelli P, Hilz M, et al. EFNS guidelines on the diagnosis and management of orthostatic hypotension. *European journal of neurology* 2006;13(9):930-6. doi: 10.1111/j.1468-1331.2006.01512.x [published Online First: 2006/08/26]

26. Herdman M, Gudex C, Lloyd A, et al. Development and preliminary testing of the new five-level version of EQ-5D (EQ-5D-5L). *Quality of life research : an international journal of quality of life aspects of treatment, care and rehabilitation* 2011;20(10):1727-36. doi: 10.1007/s11136-011-9903-x [published Online First: 2011/04/12]

27. Gallagher P, Ryan C, Byrne S, et al. STOPP (Screening Tool of Older Person's Prescriptions) and START (Screening Tool to Alert doctors to Right Treatment). Consensus validation. *International journal of clinical pharmacology and therapeutics* 2008;46(2):72-83. [published Online First: 2008/01/26]

For peer review only - http://bmjopen.bmj.com/site/about/guidelines.xhtml

28. McManus RJ, Mant J, Bray EP, et al. Telemonitoring and self-management in the control of hypertension (TASMINH2): a randomised controlled trial. *Lancet* 2010;376(9736):163-72. doi: 10.1016/s0140-6736(10)60964-6 [published Online First: 2010/07/14]

29. McManus RJ, Mant J, Haque MS, et al. Effect of self-monitoring and medication self-titration on systolic blood pressure in hypertensive patients at high risk of cardiovascular disease: the TASMIN-SR randomized clinical trial. *Jama* 2014;312(8):799-808. doi: 10.1001/jama.2014.10057 [published Online First: 2014/08/27]

30. Cuspidi C, Meani S, Lonati L, et al. Prevalence of home blood pressure measurement among selected hypertensive patients: results of a multicenter survey from six hospital outpatient hypertension clinics in Italy. *Blood pressure* 2005;14(4):251-6. doi: 10.1080/08037050500210765 [published Online First: 2005/08/30]

31. Tucker KL, Sheppard JP, Stevens R, et al. Self-monitoring of blood pressure in hypertension: A systematic review and individual patient data meta-analysis. *PLoS medicine* 2017;14(9):e1002389. doi: 10.1371/journal.pmed.1002389 [published Online First: 2017/09/20]

32. Walker E, Nowacki AS. Understanding equivalence and noninferiority testing. *Journal of general internal medicine* 2011;26(2):192-6. doi: 10.1007/s11606-010-1513-8 [published Online First: 2010/09/22]

33. Nathan A, Goodyer L, Lovejoy A, et al. 'Brown bag' medication reviews as a means of optimizing patients' use of medication and of identifying potential clinical problems. *Family practice* 1999;16(3):278-82. [published Online First: 1999/08/10]

34. Kaambwa B, Bryan S, Jowett S, et al. Telemonitoring and self-management in the control of hypertension (TASMINH2): a cost-effectiveness analysis. *European journal of preventive cardiology* 2014;21(12):1517-30. doi: 10.1177/2047487313501886 [published Online First: 2013/08/31]

35. National Guideline Centre. National Institute for Health and Care Excellence: Clinical Guidelines. Multimorbidity: Assessment, Prioritisation and Management of Care for People with Commonly Occurring Multimorbidity [NICE Guideline 56]. London: National Institute for Health and Care Excellence (UK)

Copyright (c) National Institute for Health and Care Excellence, 2016. 2016.

36. Palagyi A, Keay L, Harper J, et al. Barricades and brickwalls--a qualitative study exploring perceptions of medication use and deprescribing in long-term care. *BMC geriatrics* 2016;16:15. doi: 10.1186/s12877-016-0181-x [published Online First: 2016/01/16]

37. Potter K, Flicker L, Page A, et al. Deprescribing in Frail Older People: A Randomised Controlled Trial. *PloS one* 2016;11(3):e0149984. doi: 10.1371/journal.pone.0149984 [published Online First: 2016/03/05]

38. Sulter G, Steen C, De Keyser J. Use of the Barthel index and modified Rankin scale in acute stroke trials. *Stroke; a journal of cerebral circulation* 1999;30(8):1538-41. [published Online First: 1999/08/06]

39. Morley JE, Malmstrom TK, Miller DK. A simple frailty questionnaire (FRAIL) predicts outcomes in middle aged African Americans. *The journal of nutrition, health & aging* 2012;16(7):601-8. [published Online First: 2012/07/28]

40. Rockwood K, Song X, MacKnight C, et al. A global clinical measure of fitness and frailty in elderly people. *CMAJ : Canadian Medical Association journal = journal de l'Association medicale canadienne* 2005;173(5):489-95. doi: 10.1503/cmaj.050051 [published Online First: 2005/09/01]

For peer review only

For peer review only - http://bmjopen.bmj.com/site/about/guidelines.xhtml

41. Nasreddine ZS, Phillips NA, Bedirian V, et al. The Montreal Cognitive Assessment, MoCA: a brief screening tool for mild cognitive impairment. *Journal of the American Geriatrics Society* 2005;53(4):695-9. doi: 10.1111/j.1532-5415.2005.53221.x [published Online First: 2005/04/09]

42. Horne R, Weinman J. Patients' beliefs about prescribed medicines and their role in adherence to treatment in chronic physical illness. *Journal of psychosomatic research* 1999;47(6):555-67. [published Online First: 2000/02/08]

For peer review only

For peer review only - http://bmjopen.bmj.com/site/about/guidelines.xhtml

Tables

Table 1. Trial inclusion and exclusion criteria

**Inclusion criteria**
• Participant is willing and able to give informed consent for participation in the trial.
• Male or Female, aged 80 years or above.
• Clinic systolic blood pressure less than 150 mmHg (according to screening measurement at baseline – clinic blood pressure defined as the mean of the 2nd and 3rd readings taken at 1 minute intervals).
• Prescribed two or more antihypertensive medications to lower blood pressure for at least 12 months prior to trial entry. Antihypertensive medications defined as any ACE inhibitor, angiotensin II receptor blocker, calcium channel blocker, thiazide and thiazide-like diuretic, potassium-sparing diuretic, alpha-blocker, beta-blocker, vasodilator antihypertensives, centrally acting antihypertensives, direct renin inhibitors, adrenergic neurone blocking drugs or loop diuretics.
• Stable dose of antihypertensive medications for at least four weeks prior to trial entry.
• In the Investigator's opinion, could potentially benefit from medication reduction due to existing polypharmacy, co-morbidity, non-adherence or dislike of medicines and/or frailty
• In the Investigator's opinion, is able and willing to comply with all trial requirements.
**Exclusion criteria**
• A participant has heart failure due to LVSD and is on only ACE inhibitors/ARBs and/or beta-blockers and/or spironolactone (removing any of which would be contraindicated).
• A participant has heart failure but has not had an echocardiogram since its onset (might have undiagnosed LVSD and a compelling need for ACE inhibitors/ARB and Beta-blockers).
• Investigator deems that there is a compelling indication for antihypertensive medication continuation.
• Any other significant disease or disorder which, in the opinion of the Investigator, may either put the participants at risk because of participation in the trial, or may influence the result of the trial, or the participant's ability to participate in the trial (e.g. terminal illness, house bound and unable to attend baseline and follow up clinics).
• Suffered a myocardial infarction or stroke within the past 12 months.
• Blood pressure being managed outside of primary care.
• Unable to provide consent due to incapacity.
• A participant with secondary hypertension or previous accelerated or malignant hypertension.
• Participants who have participated in another research trial involving antihypertensive medication in the past 4 weeks.

LVSD=Left ventricular systolic dysfunction; ACE inhibitor=Angiotensin Converting Enzyme inhibitor; ARB=Angiotensin II receptor blocker

For peer review only

For peer review only - http://bmjopen.bmj.com/site/about/guidelines.xhtml

Table 2. Variables and schedule of data collection

No.	Variable	Data source		Schedule	
		Medical notes	Measured/collected at clinic	Baseline	Follow-up
1	Age		✓	✓	
2	Sex		✓	✓	
3	Ethnicity		✓	✓	
4	Marital status		✓	✓	
5	Education		✓	✓	
6	Duration of hypertension	✓		✓	
7	Past medical history	✓		✓	
8	Alcohol consumption		✓	✓	✓
9	Smoking		✓	✓	✓
10	Height		✓	✓	✓
11	Weight		✓	✓	✓
12	Clinic blood pressure (sitting and standing)		✓	✓	✓
13	Cholesterol (total and HDL)	✓		✓	
14	estimated Glomerular Filtration Rate (eGFR)	✓		✓	
15	Prescribed or over the counter medications (all medications)*	✓	✓	✓	✓
16	Quality of life (according to EQ-5D-5L)[26]		✓	✓	✓
17	Functional independence (defined by modified Rankin Scale)[38]		✓	✓	
18	Frailty (according to the FRAIL scale)[39]		✓	✓	✓
19	Frailty (according to the frailty index and electronic frailty index)[22 40]	✓	✓	✓	✓
20	Cognitive function (defined by the Montreal Cognitive Assessment [MoCA])[41]		✓	✓	
21	Adherence to medication (according to the Medication Adherence Rating Scale (MARS) Questionnaire)[42]		✓	✓	✓
22	Adherence to medication reduction		✓		✓
23	ICD-10 coded Cardiovascular events and mortality during the trial	✓			✓
24	Recording of potential side effects to medication		✓	✓	✓
25	Recording of adverse events	✓	✓		✓

HDL = High density lipoprotein; ICD = International Statistical Classification of Diseases and Related Health Problems

*Drug substance/name, formulation, dose, frequency, start date and adherence over past 12 months (according to clinical system)

For peer review only

For peer review only - http://bmjopen.bmj.com/site/about/guidelines.xhtml

Figure legends

Figure 1. Trial flow diagram

*Monitoring of blood pressure at home will be encouraged but those not willing or able will still be included in the trial. All participants will be asked to attend a safety monitoring visit with their GP/nurse four weeks after baseline.

GP = General practitioner; BP = Blood pressure; HDL = High density lipoprotein; ICD = International Statistical Classification of Diseases and Related Health Problems; CVD = Cardiovascular disease; eGFR = estimated Glomerular Filtration Rate (eGFR); MARS = Medication Adherence Rating Scale; MOCA = Montreal Cognitive Assessment

Figure 2. Medication reduction algorithm

STOPP criteria[27]

Withdraw the one of the following medications if any of the ensuing contraindications are identified:

- Thiazide diuretic with a history of gout (may exacerbate gout).
- Beta-blocker in combination with verapamil (risk of symptomatic heart block).
- Non-cardioselective beta-blocker with chronic obstructive pulmonary disease (risk of bronchospasm).
- Calcium channel blockers with chronic constipation (may exacerbate constipation).
- Use of diltiazem or verapamil with NYHA Class III or IV heart failure (may worsen heart failure).

Figure 3. Post medication reduction monitoring flow chart

Note: The full effects of most oral antihypertensives can last for up to 4-6 weeks. Frequent monitoring in the initial 4 weeks after drug withdrawal is thus not required unless BP levels are extreme or there are other clinical concerns (see above). Where systolic/diastolic BP values fall into different categories, consider the higher value. BP should be taken as the averaged second and third measurements using a validated monitor. Standard clinical care/monitoring should align with NICE recommendations.[23]

For peer review only - http://bmjopen.bmj.com/site/about/guidelines.xhtml

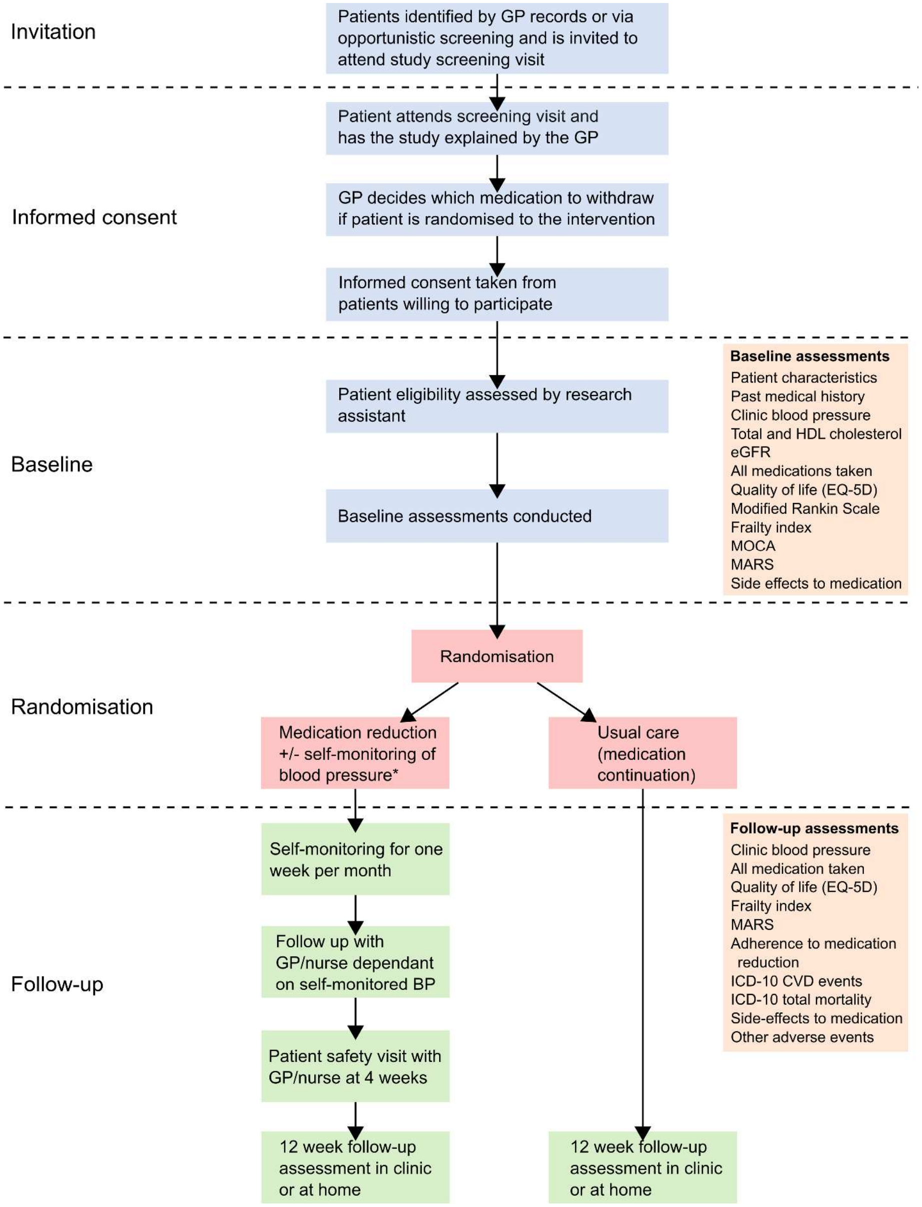
Invitation
Patients identified by GP records or via opportunistic screening and is invited to attend study screening visit
Informed consent
Patient attends screening visit and has the study explained by the GP
GP decides which medication to withdraw if patient is randomised to the intervention
Informed consent taken from patients willing to participate
Baseline
Patient eligibility assessed by research assistant
Baseline assessments conducted
Baseline assessments
Patient characteristics
Past medical history
Clinic blood pressure
Total and HDL cholesterol
eGFR
All medications taken
Quality of life (EQ-5D)
Modified Rankin Scale
Frailty index
MOCA
MARS
Side effects to medication
Randomisation
Randomisation
Medication reduction +/- self-monitoring of blood pressure*
Usual care (medication continuation)
Follow-up
Self-monitoring for one week per month
Follow up with GP/nurse dependant on self-monitored BP
Patient safety visit with GP/nurse at 4 weeks
12 week follow-up assessment in clinic or at home
12 week follow-up assessment in clinic or at home
Follow-up assessments
Clinic blood pressure
All medication taken
Quality of life (EQ-5D)
Frailty index
MARS
Adherence to medication reduction
ICD-10 CVD events
ICD-10 total mortality
Side-effects to medication
Other adverse events

Figure 1

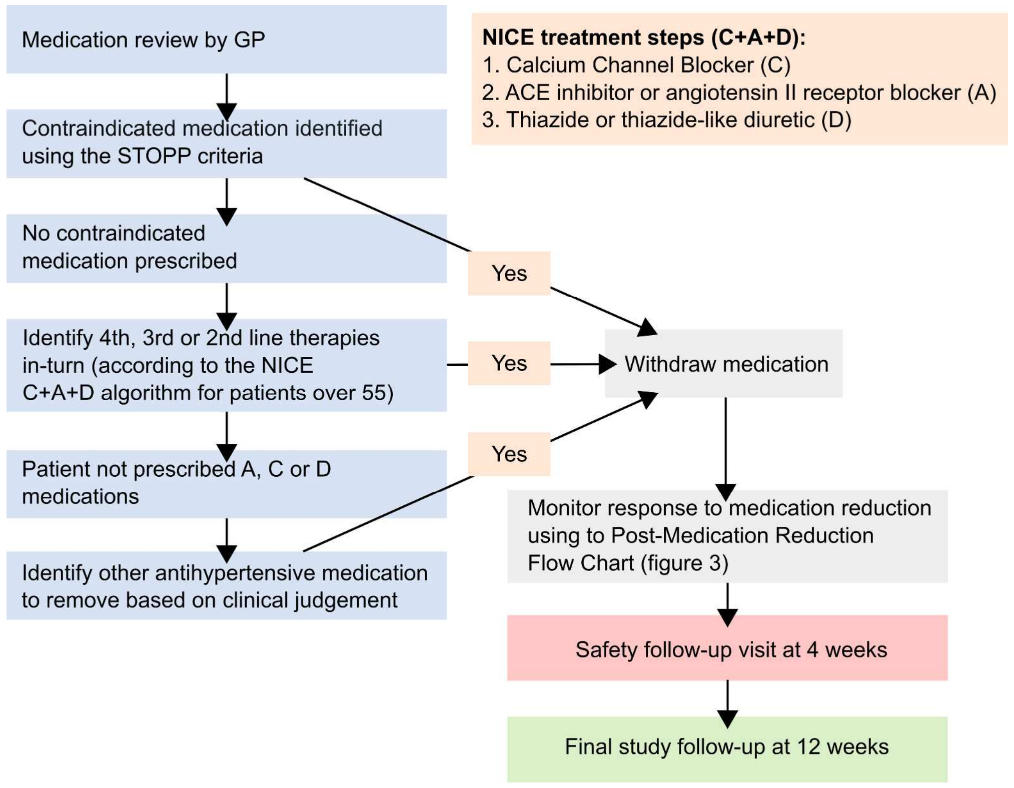

Figure 2

ew only

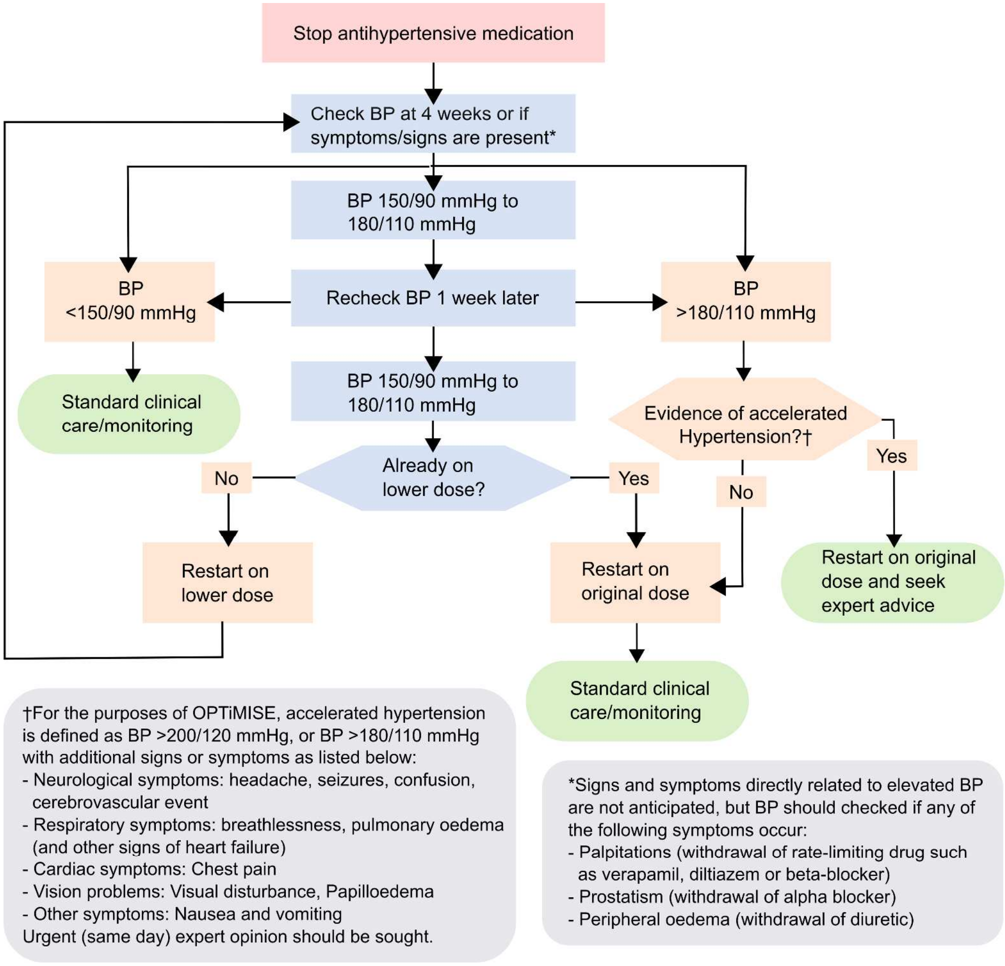
Stop antihypertensive medication
Check BP at 4 weeks or if symptoms/signs are present*
BP 150/90 mmHg to 180/110 mmHg
BP <150/90 mmHg
Recheck BP 1 week later
BP >180/110 mmHg
Standard clinical care/monitoring
BP 150/90 mmHg to 180/110 mmHg
Evidence of accelerated Hypertension?†
No
Already on lower dose?
Yes
No
Yes
Restart on lower dose
Restart on original dose
Restart on original dose and seek expert advice
Standard clinical care/monitoring
†For the purposes of OPTiMISE, accelerated hypertension is defined as BP >200/120 mmHg, or BP >180/110 mmHg with additional signs or symptoms as listed below:
- Neurological symptoms: headache, seizures, confusion, cerebrovascular event
- Respiratory symptoms: breathlessness, pulmonary oedema (and other signs of heart failure)
- Cardiac symptoms: Chest pain
- Vision problems: Visual disturbance, Papilloedema
- Other symptoms: Nausea and vomiting
Urgent (same day) expert opinion should be sought.
*Signs and symptoms directly related to elevated BP are not anticipated, but BP should checked if any of the following symptoms occur:
- Palpitations (withdrawal of rate-limiting drug such as verapamil, diltiazem or beta-blocker)
- Prostatism (withdrawal of alpha blocker)
- Peripheral oedema (withdrawal of diuretic)

Figure 3

STANDARD PROTOCOL ITEMS: RECOMMENDATIONS FOR INTERVENTIONAL TRIALS

SPIRIT 2013 Checklist: Recommended items to address in a clinical trial protocol and related documents*

Section/item	Item No	Description	Page in protocol
**Administrative information**			
Title	1	Descriptive title identifying the study design, population, interventions, and, if applicable, trial acronym	1
Trial registration	2a	Trial identifier and registry name. If not yet registered, name of intended registry	2
	2b	All items from the World Health Organization Trial Registration Data Set	n/a
Protocol version	3	Date and version identifier	n/a
Funding	4	Sources and types of financial, material, and other support	12
Roles and responsibilities	5a	Names, affiliations, and roles of protocol contributors	1, 12
	5b	Name and contact information for the trial sponsor	1
	5c	Role of study sponsor and funders, if any, in study design; collection, management, analysis, and interpretation of data; writing of the report; and the decision to submit the report for publication, including whether they will have ultimate authority over any of these activities	12
	5d	Composition, roles, and responsibilities of the coordinating centre, steering committee, endpoint adjudication committee, data management team, and other individuals or groups overseeing the trial, if applicable (see Item 21a for data monitoring committee)	12
**Introduction**			
Background and rationale	6a	Description of research question and justification for undertaking the trial, including summary of relevant studies (published and unpublished) examining benefits and harms for each intervention	3
	6b	Explanation for choice of comparators	3
Objectives	7	Specific objectives or hypotheses	3-4
Trial design	8	Description of trial design including type of trial (eg, parallel group, crossover, factorial, single group), allocation ratio, and framework (eg, superiority, equivalence, noninferiority, exploratory)	4
**Methods: Participants, interventions, and outcomes**			
Study setting	9	Description of study settings (eg, community clinic, academic hospital) and list of countries where data will be collected. Reference to where list of study sites can be obtained	4
Eligibility criteria	10	Inclusion and exclusion criteria for participants. If applicable, eligibility criteria for study centres and individuals who will	4, table 1

For peer review only - http://bmjopen.bmj.com/site/about/guidelines.xhtml

		perform the interventions (eg, surgeons, psychotherapists)	
Interventions	11a	Interventions for each group with sufficient detail to allow replication, including how and when they will be administered	5
	11b	Criteria for discontinuing or modifying allocated interventions for a given trial participant (eg, drug dose change in response to harms, participant request, or improving/worsening disease)	Figure 3
	11c	Strategies to improve adherence to intervention protocols, and any procedures for monitoring adherence (eg, drug tablet return, laboratory tests)	6-7
	11d	Relevant concomitant care and interventions that are permitted or prohibited during the trial	7
Outcomes	12	Primary, secondary, and other outcomes, including the specific measurement variable (eg, systolic blood pressure), analysis metric (eg, change from baseline, final value, time to event), method of aggregation (eg, median, proportion), and time point for each outcome. Explanation of the clinical relevance of chosen efficacy and harm outcomes is strongly recommended	4-5
Participant timeline	13	Time schedule of enrolment, interventions (including any run-ins and washouts), assessments, and visits for participants. A schematic diagram is highly recommended (see Figure)	Figure 1
Sample size	14	Estimated number of participants needed to achieve study objectives and how it was determined, including clinical and statistical assumptions supporting any sample size calculations	8
Recruitment	15	Strategies for achieving adequate participant enrolment to reach target sample size	5
**Methods: Assignment of interventions (for controlled trials)**			
Allocation:			
Sequence generation	16a	Method of generating the allocation sequence (eg, computer-generated random numbers), and list of any factors for stratification. To reduce predictability of a random sequence, details of any planned restriction (eg, blocking) should be provided in a separate document that is unavailable to those who enrol participants or assign interventions	6
Allocation concealment mechanism	16b	Mechanism of implementing the allocation sequence (eg, central telephone; sequentially numbered, opaque, sealed envelopes), describing any steps to conceal the sequence until interventions are assigned	6
Implementation	16c	Who will generate the allocation sequence, who will enrol participants, and who will assign participants to interventions	6
Blinding (masking)	17a	Who will be blinded after assignment to interventions (eg, trial participants, care providers, outcome assessors, data analysts), and how	6

For peer review only

	17b	If blinded, circumstances under which unblinding is permissible, and procedure for revealing a participant's allocated intervention during the trial	6
**Methods: Data collection, management, and analysis**			
Data collection methods	18a	Plans for assessment and collection of outcome, baseline, and other trial data, including any related processes to promote data quality (eg, duplicate measurements, training of assessors) and a description of study instruments (eg, questionnaires, laboratory tests) along with their reliability and validity, if known. Reference to where data collection forms can be found, if not in the protocol	5-7
	18b	Plans to promote participant retention and complete follow-up, including list of any outcome data to be collected for participants who discontinue or deviate from intervention protocols	7
Data management	19	Plans for data entry, coding, security, and storage, including any related processes to promote data quality (eg, double data entry; range checks for data values). Reference to where details of data management procedures can be found, if not in the protocol	6
Statistical methods	20a	Statistical methods for analysing primary and secondary outcomes. Reference to where other details of the statistical analysis plan can be found, if not in the protocol	8
	20b	Methods for any additional analyses (eg, subgroup and adjusted analyses)	8
	20c	Definition of analysis population relating to protocol non-adherence (eg, as randomised analysis), and any statistical methods to handle missing data (eg, multiple imputation)	8
**Methods: Monitoring**			
Data monitoring	21a	Composition of data monitoring committee (DMC); summary of its role and reporting structure; statement of whether it is independent from the sponsor and competing interests; and reference to where further details about its charter can be found, if not in the protocol. Alternatively, an explanation of why a DMC is not needed	9
	21b	Description of any interim analyses and stopping guidelines, including who will have access to these interim results and make the final decision to terminate the trial	n/a
Harms	22	Plans for collecting, assessing, reporting, and managing solicited and spontaneously reported adverse events and other unintended effects of trial interventions or trial conduct	9
Auditing	23	Frequency and procedures for auditing trial conduct, if any, and whether the process will be independent from investigators and the sponsor	9
**Ethics and dissemination**			
Research ethics approval	24	Plans for seeking research ethics committee/institutional review board (REC/IRB) approval	10

For peer review only

Protocol amendments	25	Plans for communicating important protocol modifications (eg, changes to eligibility criteria, outcomes, analyses) to relevant parties (eg, investigators, REC/IRBs, trial participants, trial registries, journals, regulators)	n/a
Consent or assent	26a	Who will obtain informed consent or assent from potential trial participants or authorised surrogates, and how (see Item 32)	5
	26b	Additional consent provisions for collection and use of participant data and biological specimens in ancillary studies, if applicable	n/a
Confidentiality	27	How personal information about potential and enrolled participants will be collected, shared, and maintained in order to protect confidentiality before, during, and after the trial	n/a
Declaration of interests	28	Financial and other competing interests for principal investigators for the overall trial and each study site	12
Access to data	29	Statement of who will have access to the final trial dataset, and disclosure of contractual agreements that limit such access for investigators	12
Ancillary and post-trial care	30	Provisions, if any, for ancillary and post-trial care, and for compensation to those who suffer harm from trial participation	n/a
Dissemination policy	31a	Plans for investigators and sponsor to communicate trial results to participants, healthcare professionals, the public, and other relevant groups (eg, via publication, reporting in results databases, or other data sharing arrangements), including any publication restrictions	10
	31b	Authorship eligibility guidelines and any intended use of professional writers	n/a
	31c	Plans, if any, for granting public access to the full protocol, participant-level dataset, and statistical code	n/a
**Appendices**			
Informed consent materials	32	Model consent form and other related documentation given to participants and authorised surrogates	n/a
Biological specimens	33	Plans for collection, laboratory evaluation, and storage of biological specimens for genetic or molecular analysis in the current trial and for future use in ancillary studies, if applicable	n/a

*It is strongly recommended that this checklist be read in conjunction with the SPIRIT 2013 Explanation & Elaboration for important clarification on the items. Amendments to the protocol should be tracked and dated. The SPIRIT checklist is copyrighted by the SPIRIT Group under the Creative Commons "Attribution-NonCommercial-NoDerivs 3.0 Unported" license.

For peer review only

OPtimising Treatment for MIld Systolic hypertension in the Elderly (OPTiMISE): protocol for a randomised controlled non-inferiority trial

Journal:	*BMJ Open*
Manuscript ID	bmjopen-2018-022930.R2
Article Type:	Protocol
Date Submitted by the Author:	10-Aug-2018
Complete List of Authors:	Sheppard, James; University of Oxford, Nuffield Department of Primary Care Health Sciences<br>Burt, Jenni; University of Cambridge, Cambridge Centre for Health Services Research<br>`Lown, Mark; University of Southampton<br>Temple, Eleanor; University of Oxford, Nuffield Department of Primary Care Health Sciences<br>Benson, John; University of Cambridge, GP and Primary Care Research Unit<br>Ford, Gary; Oxford University Hospitals NHS Foundation Trust, Oxford Academic Health Science Network<br>Heneghan, Carl; Oxford University, Primary Health Care<br>Hobbs, Richard; University of Oxford, Nuffield Department of Primary Care Health Sciences<br>Jowett, Sue; University of Birmingham, Health Economics Unit<br>Little, Paul; University of Southampton, Primary Care and Population Science;<br>Mant, Jonathan; University of Cambridge, General Practice and Primary Care Research Unit<br>Mollison, Jill; University of Oxford, Nuffield Department of Primary Care Health Sciences<br>Nickless, Alecia; University of Oxford, Nuffield Department of Primary Care Health Sciences<br>Ogburn, Emma; University of Oxford,<br>Payne, Rupert; University of Bristol, Centre for Academic Primary Care<br>Williams, Marney; Patient and public involvement representative<br>Yu, Ly-Mee; University of Oxford, Department of Primary Care Health Sciences<br>McManus, Richard; University of Oxford, Dept of Primary Care Health Sciences
**Primary Subject Heading**:	General practice / Family practice
Secondary Subject Heading:	Cardiovascular medicine, Geriatric medicine, Health services research
Keywords:	Multi-morbidity, Cardiovascular disease, Frailty, Antihypertensive, De-prescribing

Note: The following files were submitted by the author for peer review, but cannot be converted to PDF. You must view these files (e.g. movies) online.
OPTiMISE video inforgraphic V1.1 14.03.17.mp4

SCHOLARONE™ Manuscripts

For peer review only

James P Sheppard,[1] Jenni Burt,[2] Mark Lown,[3] Eleanor Temple,[1] John Benson,[4] Gary A Ford,[1] Carl Heneghan,[1] FD Richard Hobbs,[1] Sue Jowett,[5] Paul Little,[3] Jonathan Mant,[4] Jill Mollison,[1] Alecia Nickless,[1] Emma Ogburn,[1] Rupert Payne,[6] Marney Williams,[7] Ly-Mee Yu,[1] and Richard J McManus[1]

[1]Nuffield Department of Primary Care Health Sciences, University of Oxford, Oxford, UK
[2]The Healthcare Improvement Studies Institute, University of Cambridge, Cambridge, UK
[3]Primary Care Research Group, University of Southampton, Southampton, UK
[4]Primary Care Unit, Department of Public Health and Primary Care, University of Cambridge, Cambridge, UK
[5]Institute of Applied Health Research, University of Birmingham, Birmingham, UK
[6]Centre for Academic Primary Care, University of Bristol, Bristol, UK
[7]Patient and public involvement representative, London, UK

James P Sheppard
james.sheppard@phc.ox.ac.uk
+44 1865 617192
Nuffield Department of Primary Care Health Sciences, Radcliffe Primary Care Building, Radcliffe Observatory Quarter, University of Oxford, Oxford, OX2 6GG, UK

University of Oxford
Ms Heather House
Clinical Trials and Research Governance, Joint Research Office, Block 60, Churchill Hospital, University of Oxford, Oxford, OX3 7LE
ctrg@admin.ox.ac.uk

! 4,208 (excluding title page, abstract, references, tables and figures)
" # # 2
" # 3

For peer review only - http://bmjopen.bmj.com/site/about/guidelines.xhtml

#

Recent evidence suggests that larger blood pressure reductions and multiple antihypertensive drugs may be harmful in older people, particularly frail individuals with polypharmacy and multi-morbidity. However, there is a lack of evidence to support de-prescribing of antihypertensives, which limits the practice of medication reduction in routine clinical care. The aim of this trial is to examine whether antihypertensive medication reduction is possible in older patients without significant changes in blood pressure control at follow-up.

This trial will use a Primary Care based, open label, randomised controlled trial design. A total of 540 participants will be recruited, aged ≥80 years, with systolic blood pressure <150 mmHg and receiving ≥2 antihypertensive medications. Participants will have no compelling indication for medication continuation and will be considered to potentially benefit from medication reduction due to existing polypharmacy, co-morbidity and frailty. Following a baseline appointment, individuals will be randomised to a strategy of medication reduction (intervention) with optional self-monitoring or usual care (control). Those in the intervention group will have one antihypertensive medication stopped. The primary outcome will be to determine if a reduction in medication can achieve a proportion of participants with clinically safe blood pressure levels at 12 week follow-up (defined as a systolic blood pressure <150mmHg) which is non-inferior (within 10%) to that achieved by the usual care group. Qualitative interviews will be used to understand the barriers and facilitators to medication reduction. The study will use economic modelling to predict the long term effects of any observed changes in blood pressure and quality-of-life.

The protocol and written information has been approved by a Research Ethics Committee, medicines regulatory authority (MHRA), and national and local health research authorities. All research outputs will be published in peer-reviewed journals and presented at national and international conferences.

EudraCT 2016-004236-38; ISRCTN97503221

! 300 (max 300)

$ % Multi-morbidity, cardiovascular disease, frailty, antihypertensive, de-prescribing

For peer review only

- This will be the first UK randomised controlled trial to compare a strategy of antihypertensive medication reduction to usual care in primary care.
- The pragmatic trial design, with broad inclusion criteria, will make findings of the study externally valid in routine clinical practice.
- Allowing the attending GP to choose the medication to be reduced will maximise external validity of the trial results but precludes the possibility of blinding the participants and investigators.
- The trial will be powered to detect a non-inferior difference in blood pressure control at follow-up, but not necessarily secondary outcomes such as differences in rates of cardiovascular disease, adverse events and quality of life.

For peer review only

The general population is ageing[1] and, consequently, the number of people living with age-related chronic conditions is increasing.[2] Hypertension is the number one co-morbid condition in older people with multiple chronic conditions[3] and 52% of those aged ≥80 years are prescribed two or more antihypertensive drugs (equivalent to approximately 1.25 million people in the UK).[4] Blood pressure lowering has been shown to be effective at preventing stroke and cardiovascular disease in healthy individuals aged ≥80 years with stage 2 hypertension (systolic blood pressure of >160mmHg) and high risk stage 1 hypertension.[5 6] However, as with many trials,[7 8] these studies included healthier populations with lower polypharmacy and multi-morbidity than might be expected in the general elderly population. In addition, there is evidence to suggest that larger blood pressure reductions and multiple antihypertensive prescriptions may be harmful in older people.[9 10] Evidence from observational studies also suggests that higher intensity blood pressure treatment is associated with increased risk of falls in older people,[11] although this is also disputed.[5]

Some patients consider the increased risk of falls and other adverse events to be as important as the risk of myocardial infarction or stroke, particularly those taking medications for primary prevention of cardiovascular disease.[12] Thus, decisions over blood pressure lowering in the elderly, particularly the frail elderly, require the weighing of harms and quality of life. However, clinicians can often struggle to stop prescribing medication due to a perceived lack of evidence, fear of the reaction of other prescribers, fear of precipitating events such as stroke or angina and concern that patients will feel their care is being cut.[13 14]

There is limited evidence from randomised trials examining the safety of antihypertensive medication reduction or withdrawal.[15-19] The HYVET trial[5] enrolled some patients on antihypertensive treatment who were then randomised to placebo (effectively complete medication withdrawal) and the ANBP2 trial investigators followed up participants who withdrew medication during the trial run-in period but who were not randomised into the trial. They found younger patients with lower baseline blood pressure and fewer drug prescriptions were more likely to sustain medication withdrawal at 12 month follow-up.[20 21] However, there are few trials comparing a specified strategy of antihypertensive medication reduction with usual care in terms of effects on blood pressure control and quality of life.[17] In addition, there are no previous economic modelling studies of a strategy of medication reduction in the elderly.

The aim of this work will be to examine whether antihypertensive medication reduction in patients with controlled systolic hypertension (≤150mmHg) who are being prescribed two or more antihypertensives is possible without significant changes in blood pressure control at follow-up.

Aims and outcomes

The aim of this study is to determine whether antihypertensive medications can be safely reduced without systolic blood pressure increasing beyond what is clinical acceptable at follow-up.

The primary outcome is the proportion of participants with clinically acceptable levels at 12 week follow-up (defined as a systolic blood pressure <150mmHg) Secondary outcomes will examine:

- The proportion of participants in the intervention arm who maintain medication reduction through to follow-up (*i.e.* are *not* restarted on therapy).
- The difference in quality of life (according to EQ-5D-5L) between groups at 12 week follow-up.
- The difference in frailty (according to the frailty index)[22] between the two groups at 12 week follow-up.

For peer review only - http://bmjopen.bmj.com/site/about/guidelines.xhtml

- The difference in the change in mean clinic systolic blood pressure (from baseline) between the two groups at 12 week follow-up.
- The difference in reported side effects to medication between the two groups at 12 week follow-up (including coughs, dizziness, syncope, and ankle swelling).
- The difference in routinely reported serious adverse events between the two groups at 12 week follow-up (hospitalisation due to falls, myocardial infarction, stroke or all-cause mortality).

Design

This trial will use a Primary Care based, open label, randomised controlled, two-parallel groups, non-inferiority trial design, recruiting 540 participants with controlled blood pressure (systolic <150 mmHg) on two or more antihypertensive treatments. Participants will be randomised to a strategy of medication reduction (intervention) or usual care (control) and followed-up for 12 weeks (see figure 1). Embedded qualitative and economic analyses will examine barriers and facilitators to medication reduction and the cost effectiveness of the approach.

Trial participants

Patients eligible for the trial will be aged ≥80 years, with systolic blood pressure <150mmHg (current UK guideline recommendation)[23] receiving ≥2 antihypertensive medications. They will have no compelling indication for medication continuation and in the opinion of the attending GP, may potentially benefit from medication reduction due to existing polypharmacy, co-morbidity and/or frailty (table 1).

Participants will be identified and recruited from general practices via the UK Clinical Research Network (CRN). Potentially eligible patients will be identified by trained practice staff searching practice-based electronic disease registers using a standardised strategy. GPs will be asked to check the search results and remove people whom they believe to be unsuitable to participate in the study. Remaining potentially eligible patients will be sent letters of invitation from their GP and those expressing an interest in the trial will be asked to attend a screening and baseline appointment. Patients not responding to the first invitation will receive one reminder letter (up to four weeks after the first letter). Other potentially eligible patients may also be approached opportunistically by a member of the clinical care team. Those who do not wish to take part will be asked to fill in a short questionnaire detailing their reasons.

Baseline visit

Eligible patients will have informed consent taken by the GP. During the consent appointment, the GP will show a two-minute study video infographic (see supplementary material) and go through the participant information sheet explaining the exact nature of the trial. Having had a chance to ask questions, those individuals willing to participate will give written informed consent by means of a participant dated signature and dated signature of the GP who presented and obtained the informed consent.

Some participants will be invited to have their interview audio-recorded for qualitative analysis during their study visits. Those who are interested will be asked to sign a response slip prior to meeting the GP. Consent to audio recordings will not have a bearing on an individual’s care or eligibility for the main trial.

Those giving informed consent will be screened using the criteria in table 1 and undergo baseline measurements and randomisation by a member of the research team via participant questionnaires and a detailed notes review (table 2).

For peer review only - http://bmjopen.bmj.com/site/about/guidelines.xhtml

&' Trial inclusion and exclusion criteria

• Participant is willing and able to give informed consent for participation in the trial.
• Male or Female, aged 80 years or above.
• Clinic systolic blood pressure less than 150 mmHg (according to screening measurement at baseline – clinic blood pressure defined as the mean of the 2nd and 3rd readings taken at 1 minute intervals).
• Prescribed two or more antihypertensive medications to lower blood pressure for at least 12 months prior to trial entry. Antihypertensive medications defined as any ACE inhibitor, angiotensin II receptor blocker, calcium channel blocker, thiazide and thiazide-like diuretic, potassium-sparing diuretic, alpha-blocker, beta-blocker, vasodilator antihypertensives, centrally acting antihypertensives, direct renin inhibitors, adrenergic neurone blocking drugs or loop diuretics.
• Stable dose of antihypertensive medications for at least four weeks prior to trial entry.
• In the Investigator's opinion, could potentially benefit from medication reduction due to existing polypharmacy, co-morbidity, non-adherence or dislike of medicines and/or frailty
• In the Investigator's opinion, is able and willing to comply with all trial requirements.
(
• A participant has heart failure due to LVSD and is on only ACE inhibitors/ARBs and/or beta-blockers and/or spironolactone (removing any of which would be contraindicated).
• A participant has heart failure but has not had an echocardiogram since its onset (might have undiagnosed LVSD and a compelling need for ACE inhibitors/ARB and Beta-blockers).
• Investigator deems that there is a compelling indication for antihypertensive medication continuation.
• Any other significant disease or disorder which, in the opinion of the Investigator, may either put the participants at risk because of participation in the trial, or may influence the result of the trial, or the participant's ability to participate in the trial (e.g. terminal illness, house bound and unable to attend baseline and follow up clinics).
• Suffered a myocardial infarction or stroke within the past 12 months.
• Blood pressure being managed outside of primary care.
• Unable to provide consent due to incapacity.
• A participant with secondary hypertension or previous accelerated or malignant hypertension.
• Participants who have participated in another research trial involving antihypertensive medication in the past 4 weeks.

LVSD=Left ventricular systolic dysfunction; ACE inhibitor=Angiotensin Converting Enzyme inhibitor; ARB=Angiotensin II receptor blocker

For peer review only

For peer review only - http://bmjopen.bmj.com/site/about/guidelines.xhtml

)' Variables and schedule of data collection

" '	* #	+			
		Medical notes	Measured/collected at clinic	Baseline	Follow-up
1	Age		✓	✓	
2	Sex		✓	✓	
3	Ethnicity		✓	✓	
4	Marital status		✓	✓	
5	Education		✓	✓	
6	Duration of hypertension	✓		✓	
7	Past medical history	✓		✓	
8	Alcohol consumption		✓	✓	✓
9	Smoking		✓	✓	✓
10	Height		✓	✓	✓
11	Weight		✓	✓	✓
12	Clinic blood pressure (sitting and standing)		✓	✓	✓
13	Cholesterol (total and HDL)	✓		✓	
14	estimated Glomerular Filtration Rate (eGFR)	✓		✓	
15	Prescribed or over the counter medications (all medications)*	✓	✓	✓	✓
16	Quality of life (according to EQ-5D-5L)[24]		✓	✓	✓
17	Functional independence (defined by modified Rankin Scale)[25]		✓	✓	
18	Frailty (according to the FRAIL scale)[26]		✓	✓	✓
19	Frailty (according to the frailty index and electronic frailty index)[22 27]	✓	✓	✓	✓
20	Cognitive function (defined by the Montreal Cognitive Assessment [MoCA])[28]		✓	✓	
21	Adherence to medication (according to the Medication Adherence Rating Scale (MARS) Questionnaire)[29]		✓	✓	✓
22	Adherence to medication reduction		✓		✓
23	ICD-10 coded Cardiovascular events and mortality during the trial	✓			✓
24	Recording of potential side effects to medication		✓	✓	✓
25	Recording of adverse events	✓	✓		✓

HDL = High density lipoprotein; ICD = International Statistical Classification of Diseases and Related Health Problems

*Drug substance/name, formulation, dose, frequency, start date and adherence over past 12 months (according to clinical system)

For peer review only

For peer review only - http://bmjopen.bmj.com/site/about/guidelines.xhtml

Blood pressure will be measured in a standardised fashion using the clinically validated[30] BpTRU blood pressure monitor which automatically records six blood pressure measurements at one minute intervals. Blood pressure readings will be taken in the left arm (where appropriate) after participants have been seated for at least five minutes of rest, using an appropriate sized cuff. The mean of the 2nd and 3rd readings will be used to define the primary outcome. To test for orthostatic hypotension, two further readings will be taken in the standing position after one and three minutes.[31] Only the research facilitator/nurse will be present during the blood pressure measurements. Orthostatic hypotension will be defined as a $\geq$20mmHg drop in systolic blood pressure within three minutes of standing.

All data will be collected via an electronic case report form (eCRF) linked to the study database. Participants will be given the option to enter responses to questionnaires themselves or with assistance from the research team. Where questionnaires are not validated for use on a tablet computer,[24] or where individuals are not comfortable using one, paper copies will be made available for completion.

Randomisation

Consenting participants will be individually randomised (1:1 allocation ratio) to one of two study arms using a fully validated web-based system (Sortition®) with manual telephone back up. Participants will not be randomised until after consent has been taken and baseline assessments have been completed. A computer generated non-deterministic algorithm, minimising on practice and baseline systolic blood pressure will be used to ensure these covariates are balanced between the two intervention arms.

The study will use an open label design, so patients and practitioners will not be blinded to the intervention or study endpoints. Therefore, codebreaking will not be necessary. The statistical analysis will be performed blind to patient allocation.

Intervention group

Participating GPs will review each participant's antihypertensive medication regimen prior to the baseline appointment, and decide which medication should be removed if they are randomised to the intervention arm of the trial. The choice of medication to be withdrawn will be at the discretion of the GP, but their decision will be informed by an individual's co-morbidities and existing guidelines, where appropriate (figure 2). Specifically, participating GPs will be encouraged to identify previously unrecognised contraindications to medication, defined by the STOPP criteria.[32] In the absence of these, or a strong clinical rationale for continuing despite a STOPP criteria being met, GPs will be recommended to reduce antihypertensive medications in reverse of the NICE C+A+D algorithm for older patients (figure 2).[23] All participants in the trial will remain on at least one antihypertensive.

Once a medication has been removed, GPs or other appropriate, delegated healthcare professionals will closely monitor the participant's response to medication reduction: they will be given advice about what and when to monitor (figure 3), but this schedule will be flexible. All participants will be expected to return for at least one routine safety follow-up visit, and further visits may be required if blood pressure is raised (≥150 mmHg), or adverse events occur. Where blood pressure is persistently raised, GPs will be expected to re-adjust medication (dose or type), rendering the likelihood of a serious adverse event occurring as a result of the intervention very low.

Self-monitoring

For peer review only - http://bmjopen.bmj.com/site/about/guidelines.xhtml

All participants randomised to the medication reduction arm of the trial will be given the option to self-monitor their blood pressure at home. Those accepting will be trained using protocols developed in the previous TASMIN trials[33 34] and will be given simple and clear instructions to contact their GP if their blood pressure rises above what is considered clinically safe (e.g. home systolic blood pressure >145mmHg on all readings taken in a week). Participants will be advised to self-monitor (or have a carer monitor) at least 4 times per week in the last week of each month of follow-up (weeks 4, 8 and 12), although they can monitor more frequently if they wish. Differential use of self-monitoring in the intervention group, or indeed in the control group (many patients now self-monitor routinely)[35] is not expected to impact on the study results, since there is good evidence that self-monitoring only affects blood pressure levels if used in combination with a co-intervention.[36] All other clinical care will continue as usual.

Control group

Those allocated to the control arm of the study will continue usual clinical care (i.e. they will continue to take antihypertensive medications as prescribed and will not self-monitor unless already doing so). No other medication changes will be mandated and participating GPs will be asked to manage all other care according to usual clinical practice.

Follow-up visits

Participants will attend one research follow-up clinic, 12 weeks (±2 weeks) after baseline and those in the intervention will attend one additional safety visit after four weeks (±2 weeks) (figure 1). A period of four weeks is expected to be sufficiently long enough to assess the impact of antihypertensive medication reduction, since these drugs usually take approximately four weeks to 'wash out' of a patient's system. Earlier safety visits are not recommended since they could provide false reassurance that blood pressure is within safe limits if the withdrawn drug has not washed out of the participant's system.

The follow-up assessments will include standardised blood pressure measurement (for assessment of the primary outcome), questionnaire assessments and adherence to the trial medication regime, side effects and adverse events (table 2). Where possible, all participants will be flagged for mortality and hospital admissions using NHS patient tracking services, permitting long-term follow-up for up to 5 years after the trial has finished.

Each participant has the right to withdraw from the trial at any time. We will ask all participants to attend a follow-up visit as far as is practicable, regardless of whether medication is re-introduced to participants in the intervention group, or a participant in the control group has medication withdrawn. Unless a participant withdraws consent, vital status will be assessed even where an individual has been lost to follow-up (for instance moved away). If given, the reason for withdrawal will be recorded in the eCRF.

Internal feasibility study

A two-stage internal feasibility study will be conducted to examine methods of patient invitation and rates of recruitment, before proceeding with the main trial. The first feasibility phase will last for a minimum of three months and aim to recruit approximately 25 participants from a minimum of 3-5 practices. The aim will be to establish whether or not anyone will be willing to participate in the study.

The second feasibility phase will focus on recruitment rates for the main trial and whether the intended sample size is likely to be met during the recruitment period. This phase will have a recruitment target of 75 participants from ten practices over 6 months, giving a total sample for the feasibility study of 100

For peer review only - http://bmjopen.bmj.com/site/about/guidelines.xhtml

participants. A recruitment rate of 15% of invitations sent is expected. The following actions will be considered to address varying rates of recruitment at the end of the feasibility phases:

- If ≥100 participants are recruited – trial will proceed as planned
- If 75-99 participants are recruited – recruitment materials/method will be re-examined and edited where necessary following discussions with stakeholders and patient and public involvement representatives.
- If 50-74 participants are recruited – the allocation of resources and recruitment criteria will be re-examined using information gathered from concurrent qualitative work.
- If <50 participants are recruited – the Trial Steering Committee (TSC) will decide, in discussion with the Data Monitoring and Ethics Committee (DMEC) and the funders, whether the trial should be stopped due to futility.

Sample size calculation

Assuming that 100% of participants in the usual care group, and 96% of those in the medication reduction group have controlled systolic blood pressure levels (<150mmHg) at follow-up, approximately 540 participants will be required to detect a non-inferior difference in systolic blood pressure control between groups. Calculations assume a 10% non-inferiority margin, 90% power, 2.5% 1-sided level of significance, 10% loss to follow-up and a 10% dilution effect due to cross-over between arms. There is no existing precedent for an appropriate margin of non-inferiority in this type of trial, but 10% was deemed useful to inform future doctor-patient discussions about medication reduction: if the non-inferiority margin is met, it will suggest that for every ten patients who have their medication reduced, at least nine will still have controlled blood pressure at 12 week follow-up.

Statistical analysis

A detailed statistical analysis plan will be agreed prior to the end of the trial. The primary and secondary analyses will be by intention to treat (ITT), unless stated otherwise. The primary analysis will be a non-inferiority analysis by means of the “two one-sided test” (TOST) procedure,[37] whereby the 95% confidence interval for the relative risk of participants with systolic blood pressure at 12 weeks below 150 mmHg between the medication reduction group and the usual care group is calculated. This will be obtained by means of a generalised linear mixed effects model with GP surgery included as a random effect and baseline blood pressure as a fixed effect. If the lower limit of the confidence interval is more than 0.9 (equal to a risk difference of 10%) then the research hypothesis that medication reduction will be non-inferior in terms of blood pressure control to usual care will be accepted. As a secondary analysis of the primary outcome, a per-protocol (PP) analysis will be performed, since ITT can be anticonservative for a non-inferiority hypothesis.[37] Participants who received the medication reduction intervention in the PP analysis will be defined as a participant in the medication reduction arm who maintained their medication reduction throughout the 12 week follow-up period.

Secondary analyses will examine the proportion of participants in the medication reduction arm who maintained their medication reduction throughout the 12 week follow-up period. Secondary outcomes will be analysed by means of linear mixed effects models, adjusting for the baseline level of the outcome and baseline systolic blood pressure and including practice as a random effect: systolic blood pressure, EQ-5D-5L and the Frailty index/frail scale. The difference in the rate of side effects and adverse events between the medication reduction and usual care arms will be analysed by means of a logistic mixed effects model adjusting for baseline systolic blood pressure and including practice as a random effect.

For peer review only - http://bmjopen.bmj.com/site/about/guidelines.xhtml

Exploratory subgroup analyses of blood pressure control, change in blood pressure and maintenance of medication reduction will be conducted by different levels of baseline frailty, functional independence, cognitive function, number of medications prescribed at baseline and number of co-morbidities at baseline.

, ,

This protocol was developed through discussions with older patients and carers and members of an AgeUK day centre. MW is a stroke survivor with experience as a volunteer on the elderly ward of Charing Cross Hospital. She was consulted on the suitability and design of the trial and is a member of the trial management group. Methods of patient approach, including the design of the video infographic, patient information sheet and consent form were all reviewed by patient representatives prior to formal approval. The TSC includes two independent patient representatives responsible for overseeing the conduct of the trial.

Adverse events that are observed by an investigator or reported by the participant will be recorded on the AE log at any time during the study but AEs will be specifically asked about at the 12 week follow up. Serious adverse events (SAEs) will be reported to the coordinating centre within 24 hours of discovery or notification of the event. All SAE reports will be reviewed by the DMEC chair on a monthly basis, and by the full DMEC at meetings held every 6 months. The DMEC will include a geriatrician, statistician and consultant clinical pharmacologist. They will be responsible for safeguarding trial participants, monitoring emerging trial data including identifying any trends, such as increases in unexpected events, and take appropriate action where necessary.

All adverse events labelled possibly, probably or definitely related will be considered as related to the intervention. Since there are no sections of the Summary of medicinal Product Characteristics, or previous clinical studies which detail expected adverse events as a result of medication withdrawal, all SAEs at least possibly related, and not as a result of re-introduction of withdrawn medication, will be considered unexpected and reported as SUSARs. Fatal and life-threatening SUSARs will be reported by the chief investigator to the relevant Competent Authority and Research Ethics Committee no later than 7 calendar days after the Sponsor or delegate is first aware of the reaction. All other SUSARs will be reported within 15 calendar days.

- , #

Study 1: interviews with doctors and patient

Face-to-face interviews with GPs and patients will be conducted to generate understanding about the barriers and facilitators to antihypertensive medication reduction. Informed consent will be sought from approximately 15 GPs to provide a broad range of opinion from varying practice sizes (small to large) and settings (rural to urban). Participating GPs will also be asked to identify up to 15 patients for interview, based on the same inclusion criteria as those applied to participants in the main trial.

Interviews with GPs will use a chart-stimulated recall approach to explore the factors which influence their treatment choices in older hypertensive patients. Anonymised electronic health records will be used to focus discussions about how GPs would feel about reducing antihypertensive medications. Interviews with patients will use 'brown bag' medication review techniques[38] to create a complete record of medication held, with a commentary on usage from the participants' perspective. Diagrammatic elicitation techniques will be used to complete a relational map outlining participants' circumstances and how these relate to the

For peer review only

medications taken. These sketches will be used as the basis for a discussion on the implications of withdrawing antihypertensive medications, and what this "gap" might mean for the patient.

All interviews will be transcribed verbatim, stored and organised using NVivo software (QSR International Pty Ltd, Doncaster, Victoria, Australia). Interview and visual data from GP and patient interviews will be subjected to thematic analysis, with a particular orientation to exploring clinical and patient perspectives on the barriers and facilitators to reducing antihypertensives.

Study 2: Assessment of trial recruitment and data collection procedures

The aim of the second qualitative study will be to explore how information is presented within recruitment appointments and how this might impact on consent to participate, with a view to ensuring robust trial procedures using an iterative process. This will be achieved by audio-recording (with consent) up to 75 consultations between GPs or research assistants and eligible patients.

Thematic analysis will be undertaken on a sample of around 15-20 consultations comprising patients who did, or did not consent to participate, to consider (a) terminology used, (b) presentation of the de-prescribing approach and (c) presentation of randomisation. This will inform on-going trial procedures and future implementation.

#

This work will adapt a previous decision-analytic model examining the long-term costs and benefits from blood pressure lowering treatment[39] to include potential harms of treatment. The model will be adjusted for the effects of blood pressure lowering on cardiovascular disease risk, costs and quality-adjusted-life years (QALYs) to match the older population involved in this work. Costs of the therapies prescribed, side-effects and acute and long term costs of cardiovascular events will be obtained within the trial and from the literature. Quality of life on each treatment strategy will be obtained from the trial data using EQ-5D-5L, and previous studies will inform utility values for cardiovascular disease health states and the impact of side effects. The model will determine the cost per additional QALY gained of the medication reduction intervention versus usual care and analyses will be conducted from a health and social services perspective. The model will be run with a lifetime perspective, with costs and benefits discounted at a rate of 3.5%. A value of information analysis will assess whether a further trial would be appropriate to reduce decision uncertainty, and identify those parameters where more precise estimates would be most valuable and should therefore be chosen as outcomes for such a trial.

This research involves older participants, some of whom may be considered vulnerable. Great care will be taken to ensure all potential participants have the trial clearly explained, and are given sufficient time to decide whether to give informed consent. This will include provision of simplified, participant information sheets with large fonts, video infographics to explain the study and extended GP consultation periods for explaining the study and taking informed consent. The protocol, informed consent form, participant information sheet and all other participant facing material have been approved by the Research Ethics Committee (South Central - Oxford A; ref 16/SC/0628), Medicines and Healthcare products Regulatory Agency (ref 21584/0371/001-0001), host institution(s) and Health Research Authority. The study sponsor reviewed and ensured all indemnity and insurance requirements for the trial were in place prior to the start of recruitment.

For peer review only

For peer review only - http://bmjopen.bmj.com/site/about/guidelines.xhtml

All research outputs from this work will be published in peer-reviewed journals, presented at scientific conferences and lay and social media (e.g. Twitter, blogs). 'Patient friendly' study summary documents and infographics will be made available to all participants at the end of the trial via the study website.

Current trial status

The trial commenced recruitment on 10th April 2017 and is estimated to continue recruitment until September 2018.

+

Current guidelines in the UK suggest that doctors should ensure that patients are fully informed of the benefits and risks of their prescribed medications and where appropriate, discuss the potential for medication withdrawal in frail individuals with multi-morbidity. [40] This is difficult given consultation time constraints and fear that de-prescribing might result in harm.[41] This is compounded by conflicting and inconclusive evidence about the benefits and harms of treatment, and a lack of evidence about what will happen if these treatments are reduced.

The ECSTATIC trial enrolled 1,067 younger participants aged 40-70 years, taking antihypertensives for primary prevention of cardiovascular disease.[18] The trial demonstrated that only 27% of participants were able to maintain medication reduction throughout follow-up and at 3 months, systolic blood pressure was on average 6 mmHg higher in the de-prescribing group. At 2 year follow-up, the risk of uncontrolled blood pressure was significantly higher in those patients attempting to de-prescribe. Unlike the present study, the medication reduction algorithm used did not encourage reintroduction of therapy if blood pressure was persistently raised.

The DANTE study[16] examined the effect of complete antihypertensive medication discontinuation in 385 patients over the age of 75 years and with mild cognitive deficits. After 16 weeks of follow-up, they observed a 7/3 mmHg increase in blood pressure but no difference in overall cognition compound score or quality of life between groups. A study by Van der Wardt and colleagues[19] examined the feasibility trial reducing antihypertensives in patients with dementia, but was only able to recruit 9 participants for the withdrawal programme (1% recruitment rate) and a larger trial was deemed unfeasible. Similarly, the OPTIMED trial[42] demonstrated in 95 participants that a broader de-prescribing approach is achievable in patients living in nursing homes, but was unable to examine the effect on clinical outcomes due to recruitment issues resulting on only 38% of the planned sample size being enrolled.

The OPTiMISE trial will target frail individuals with polypharmacy and co-morbidity, and aim to establish whether a strategy of antihypertensive medication reduction is safe and acceptable to older patients. The findings of this trial will support better patient-centred management plans for the prevention of cardiovascular disease in older individuals and inform future de-prescribing trials in primary care.

For peer review only - http://bmjopen.bmj.com/site/about/guidelines.xhtml

. %

The authors acknowledge the support of the Primary Care Clinical Trials Unit, staff from the NIHR CRNs including Thames Valley and South Midlands, Cambridge, Southampton, West Midlands (Central and South) and West of England, and Lucy Curtin for administrative support. Margaret Ogden and Anita Higham serve as patient representatives for the trial steering committee. Additional members of the trial steering committee are Prof Tom Robinson (chair), Prof Rod Taylor and Dr Peter Bower. Members of the data monitoring committee are Prof John Gladman (chair), Prof Una Martin and Dr Martyn Lewis. The sponsor and funder had no role in the study design, writing of the paper; or the decision to submit this protocol for publication, which was made jointly by the authors who have all approved the final manuscript. Finally, this work would not be possible without the support of the participating practices and participants.

/

This work receives joint funding from the National Institute for Health Research (NIHR) Oxford Collaboration for Leadership in Applied Health Research and Care (CLAHRC) at Oxford Health NHS Foundation Trust (ref: P2-501) and the NIHR School for Primary Care Research (SPCR; ref 335). JS and RJMcM have been funded by an NIHR Professorship (NIHR-RP-R2-12-015). FDRH acknowledges part support from the NIHR SPCR, the NIHR CLAHRC Oxford, and the NIHR Oxford Biomedical Research Centre (BRC). CH receives support from the NIHR SPCR and NIHR Oxford BRC. The views and opinions expressed are those of the authors and do not necessarily reflect those of the NHS, NIHR, or the Department of Health.

The authors declare no conflicts of interest.

0 #

JS conceived, designed and secured funding for the study with JBu, ML, JBe, GF, CH, FDRH, SJ, PL, JM, EO, RP, MW, LMY and RJMcM. JS wrote the first draft. AN, JMo and LMY provided the sample size calculations and statistical analysis section. JBu provided the qualitative section. SJ provided the health economic section. All authors reviewed and edited the manuscript. ET is the trial manager. JS and RJMcM are co-chief investigators and will act as guarantors for this work.

+

Data sharing requests will be considered by the corresponding author.

For peer review only

For peer review only - http://bmjopen.bmj.com/site/about/guidelines.xhtml

1

1. Office for National Statistics. Mid-year populations estimates: Aging, fastest increase in the 'oldest old'. http://www.ons.gov.uk, 2010.

2. Barnett K, Mercer SW, Norbury M, et al. Epidemiology of multimorbidity and implications for health care, research, and medical education: a cross-sectional study. *Lancet* 2012;380(9836):37-43. doi: 10.1016/s0140-6736(12)60240-2 [published Online First: 2012/05/15]

3. Violan C, Foguet-Boreu Q, Roso-Llorach A, et al. Burden of multimorbidity, socioeconomic status and use of health services across stages of life in urban areas: a cross-sectional study. *BMC public health* 2014;14:530. doi: 10.1186/1471-2458-14-530 [published Online First: 2014/06/03]

4. Sheppard JP, Singh S, Fletcher K, et al. Impact of age and sex on primary preventive treatment for cardiovascular disease in the West Midlands, UK: cross sectional study. *BMJ (Clinical research ed)* 2012;345:e4535. doi: 10.1136/bmj.e4535 [published Online First: 2012/07/14]

5. Beckett NS, Peters R, Fletcher AE, et al. Treatment of hypertension in patients 80 years of age or older. *The New England journal of medicine* 2008;358(18):1887-98. doi: 10.1056/NEJMoa0801369 [published Online First: 2008/04/02]

6. A Randomized Trial of Intensive versus Standard Blood-Pressure Control. *The New England journal of medicine* 2015 doi: 10.1056/NEJMoa1511939 [published Online First: 2015/11/10]

7. McKee M, Britton A, Black N, et al. Interpreting the evidence: choosing between randomised and non-randomised studies. *BMJ (Clinical research ed)* 1999;319(7205):312-5.

8. van Deudekom FJ, Postmus I, van der Ham DJ, et al. External validity of randomized controlled trials in older adults, a systematic review. *PloS one* 2017;12(3):e0174053. doi: 10.1371/journal.pone.0174053 [published Online First: 2017/03/28]

9. Bejan-Angoulvant T, Saadatian-Elahi M, Wright JM, et al. Treatment of hypertension in patients 80 years and older: the lower the better? A meta-analysis of randomized controlled trials. *Journal of hypertension* 2010;28(7):1366-72. doi: 10.1097/HJH.0b013e328339f9c5 [published Online First: 2010/06/25]

10. Benetos A, Labat C, Rossignol P, et al. Treatment With Multiple Blood Pressure Medications, Achieved Blood Pressure, and Mortality in Older Nursing Home Residents: The PARTAGE Study. *JAMA internal medicine* 2015 doi: 10.1001/jamainternmed.2014.8012 [published Online First: 2015/02/17]

11. Tinetti ME, Han L, Lee DS, et al. Antihypertensive medications and serious fall injuries in a nationally representative sample of older adults. *JAMA internal medicine* 2014;174(4):588-95. doi: 10.1001/jamainternmed.2013.14764 [published Online First: 2014/02/26]

12. Fried TR, Tinetti ME, Towle V, et al. Effects of benefits and harms on older persons' willingness to take medication for primary cardiovascular prevention. *Archives of internal medicine* 2011;171(10):923-8. doi: 10.1001/archinternmed.2011.32 [published Online First: 2011/03/02]

13. Schuling J, Gebben H, Veehof LJ, et al. Deprescribing medication in very elderly patients with multimorbidity: the view of Dutch GPs. A qualitative study. *BMC family practice* 2012;13:56. doi: 10.1186/1471-2296-13-56 [published Online First: 2012/06/16]

14. Scott IA, Anderson K, Freeman CR, et al. First do no harm: a real need to deprescribe in older patients. *The Medical journal of Australia* 2014;201(7):390-2. [published Online First: 2014/10/09]

For peer review only - http://bmjopen.bmj.com/site/about/guidelines.xhtml

15. Iyer S, Naganathan V, McLachlan AJ, et al. Medication withdrawal trials in people aged 65 years and older: a systematic review. *Drugs & aging* 2008;25(12):1021-31. doi: 10.2165/0002512-200825120-00004 [published Online First: 2008/11/22]

16. Moonen JE, Foster-Dingley JC, de Ruijter W, et al. Effect of Discontinuation of Antihypertensive Treatment in Elderly People on Cognitive Functioning--the DANTE Study Leiden: A Randomized Clinical Trial. *JAMA internal medicine* 2015;175(10):1622-30. doi: 10.1001/jamainternmed.2015.4103 [published Online First: 2015/08/25]

17. van der Wardt V, Harrison JK, Welsh T, et al. Withdrawal of antihypertensive medication: a systematic review. *Journal of hypertension* 2017;35(9):1742-49. doi: 10.1097/hjh.0000000000001405 [published Online First: 2017/05/10]

18. Luymes CH, Poortvliet RKE, van Geloven N, et al. Deprescribing preventive cardiovascular medication in patients with predicted low cardiovascular disease risk in general practice - the ECSTATIC study: a cluster randomised non-inferiority trial. *BMC medicine* 2018;16(1):5. doi: 10.1186/s12916-017-0988-0 [published Online First: 2018/01/13]

19. van der Wardt V, Burton JK, Conroy S, et al. Withdrawal of antihypertensive therapy in people with dementia: feasibility study. *Pilot and feasibility studies* 2018;4:29. doi: 10.1186/s40814-017-0221-0 [published Online First: 2018/01/18]

20. Nelson MR, Reid CM, Krum H, et al. Predictors of normotension on withdrawal of antihypertensive drugs in elderly patients: prospective study in second Australian national blood pressure study cohort. *BMJ (Clinical research ed)* 2002;325(7368):815. [published Online First: 2002/10/12]

21. Nelson MR, Reid CM, Krum H, et al. Short-term predictors of maintenance of normotension after withdrawal of antihypertensive drugs in the second Australian National Blood Pressure Study (ANBP2). *American journal of hypertension* 2003;16(1):39-45. [published Online First: 2003/01/09]

22. Clegg A, Bates C, Young J, et al. Development and validation of an electronic frailty index using routine primary care electronic health record data. *Age and ageing* 2016;45(3):353-60. doi: 10.1093/ageing/afw039 [published Online First: 2016/03/06]

23. National Clinical Guideline C. National Institute for Health and Clinical Excellence Guidance CG127. Hypertension: The Clinical Management of Primary Hypertension in Adults: Update of Clinical Guidelines 18 and 34. London: Royal College of Physicians (UK) 2011.

24. Herdman M, Gudex C, Lloyd A, et al. Development and preliminary testing of the new five-level version of EQ-5D (EQ-5D-5L). *Quality of life research : an international journal of quality of life aspects of treatment, care and rehabilitation* 2011;20(10):1727-36. doi: 10.1007/s11136-011-9903-x [published Online First: 2011/04/12]

25. Sulter G, Steen C, De Keyser J. Use of the Barthel index and modified Rankin scale in acute stroke trials. *Stroke; a journal of cerebral circulation* 1999;30(8):1538-41. [published Online First: 1999/08/06]

26. Morley JE, Malmstrom TK, Miller DK. A simple frailty questionnaire (FRAIL) predicts outcomes in middle aged African Americans. *The journal of nutrition, health & aging* 2012;16(7):601-8. [published Online First: 2012/07/28]

27. Rockwood K, Song X, MacKnight C, et al. A global clinical measure of fitness and frailty in elderly people. *CMAJ : Canadian Medical Association journal = journal de l'Association medicale canadienne* 2005;173(5):489-95. doi: 10.1503/cmaj.050051 [published Online First: 2005/09/01]

For peer review only

For peer review only - http://bmjopen.bmj.com/site/about/guidelines.xhtml

28. Nasreddine ZS, Phillips NA, Bedirian V, et al. The Montreal Cognitive Assessment, MoCA: a brief screening tool for mild cognitive impairment. *Journal of the American Geriatrics Society* 2005;53(4):695-9. doi: 10.1111/j.1532-5415.2005.53221.x [published Online First: 2005/04/09]

29. Horne R, Weinman J. Patients' beliefs about prescribed medicines and their role in adherence to treatment in chronic physical illness. *Journal of psychosomatic research* 1999;47(6):555-67. [published Online First: 2000/02/08]

30. Mattu GS, Heran BS, Wright JM. Overall accuracy of the BpTRU--an automated electronic blood pressure device. *Blood pressure monitoring* 2004;9(1):47-52. [published Online First: 2004/03/17]

31. Lahrmann H, Cortelli P, Hilz M, et al. EFNS guidelines on the diagnosis and management of orthostatic hypotension. *European journal of neurology* 2006;13(9):930-6. doi: 10.1111/j.1468-1331.2006.01512.x [published Online First: 2006/08/26]

32. Gallagher P, Ryan C, Byrne S, et al. STOPP (Screening Tool of Older Person's Prescriptions) and START (Screening Tool to Alert doctors to Right Treatment). Consensus validation. *International journal of clinical pharmacology and therapeutics* 2008;46(2):72-83. [published Online First: 2008/01/26]

33. McManus RJ, Mant J, Bray EP, et al. Telemonitoring and self-management in the control of hypertension (TASMINH2): a randomised controlled trial. *Lancet* 2010;376(9736):163-72. doi: 10.1016/s0140-6736(10)60964-6 [published Online First: 2010/07/14]

34. McManus RJ, Mant J, Haque MS, et al. Effect of self-monitoring and medication self-titration on systolic blood pressure in hypertensive patients at high risk of cardiovascular disease: the TASMIN-SR randomized clinical trial. *Jama* 2014;312(8):799-808. doi: 10.1001/jama.2014.10057 [published Online First: 2014/08/27]

35. Cuspidi C, Meani S, Lonati L, et al. Prevalence of home blood pressure measurement among selected hypertensive patients: results of a multicenter survey from six hospital outpatient hypertension clinics in Italy. *Blood pressure* 2005;14(4):251-6. doi: 10.1080/08037050500210765 [published Online First: 2005/08/30]

36. Tucker KL, Sheppard JP, Stevens R, et al. Self-monitoring of blood pressure in hypertension: A systematic review and individual patient data meta-analysis. *PLoS medicine* 2017;14(9):e1002389. doi: 10.1371/journal.pmed.1002389 [published Online First: 2017/09/20]

37. Walker E, Nowacki AS. Understanding equivalence and noninferiority testing. *Journal of general internal medicine* 2011;26(2):192-6. doi: 10.1007/s11606-010-1513-8 [published Online First: 2010/09/22]

38. Nathan A, Goodyer L, Lovejoy A, et al. 'Brown bag' medication reviews as a means of optimizing patients' use of medication and of identifying potential clinical problems. *Family practice* 1999;16(3):278-82. [published Online First: 1999/08/10]

39. Kaambwa B, Bryan S, Jowett S, et al. Telemonitoring and self-management in the control of hypertension (TASMINH2): a cost-effectiveness analysis. *European journal of preventive cardiology* 2014;21(12):1517-30. doi: 10.1177/2047487313501886 [published Online First: 2013/08/31]

40. National Guideline Centre. National Institute for Health and Care Excellence: Clinical Guidelines. Multimorbidity: Assessment, Prioritisation and Management of Care for People with Commonly Occurring Multimorbidity [NICE Guideline 56]. London: National Institute for Health and Care Excellence (UK)

Copyright (c) National Institute for Health and Care Excellence, 2016. 2016.

For peer review only

For peer review only - http://bmjopen.bmj.com/site/about/guidelines.xhtml

41. Palagyi A, Keay L, Harper J, et al. Barricades and brickwalls--a qualitative study exploring perceptions of medication use and deprescribing in long-term care. *BMC geriatrics* 2016;16:15. doi: 10.1186/s12877-016-0181-x [published Online First: 2016/01/16]

42. Potter K, Flicker L, Page A, et al. Deprescribing in Frail Older People: A Randomised Controlled Trial. *PloS one* 2016;11(3):e0149984. doi: 10.1371/journal.pone.0149984 [published Online First: 2016/03/05]

For peer review only

For peer review only - http://bmjopen.bmj.com/site/about/guidelines.xhtml

/

/ &' Trial flow diagram

*Monitoring of blood pressure at home will be encouraged but those not willing or able will still be included in the trial. All participants will be asked to attend a safety monitoring visit with their GP/nurse four weeks after baseline.

GP = General practitioner; BP = Blood pressure; HDL = High density lipoprotein; ICD = International Statistical Classification of Diseases and Related Health Problems; CVD = Cardiovascular disease; eGFR = estimated Glomerular Filtration Rate (eGFR); MARS = Medication Adherence Rating Scale; MOCA = Montreal Cognitive Assessment

/)' Medication reduction algorithm

Withdraw the one of the following medications if any of the ensuing contraindications are identified:

- Thiazide diuretic with a history of gout (may exacerbate gout).
- Beta-blocker in combination with verapamil (risk of symptomatic heart block).
- Non-cardioselective beta-blocker with chronic obstructive pulmonary disease (risk of bronchospasm).
- Calcium channel blockers with chronic constipation (may exacerbate constipation).
- Use of diltiazem or verapamil with NYHA Class III or IV heart failure (may worsen heart failure).

/ 2' Post medication reduction monitoring flow chart

Note: The full effects of most oral antihypertensives can last for up to 4-6 weeks. Frequent monitoring in the initial 4 weeks after drug withdrawal is thus not required unless BP levels are extreme or there are other clinical concerns (see above). Where systolic/diastolic BP values fall into different categories, consider the higher value. BP should be taken as the averaged second and third measurements using a validated monitor. Standard clinical care/monitoring should align with NICE recommendations.[23]

For peer review only

For peer review only - http://bmjopen.bmj.com/site/about/guidelines.xhtml

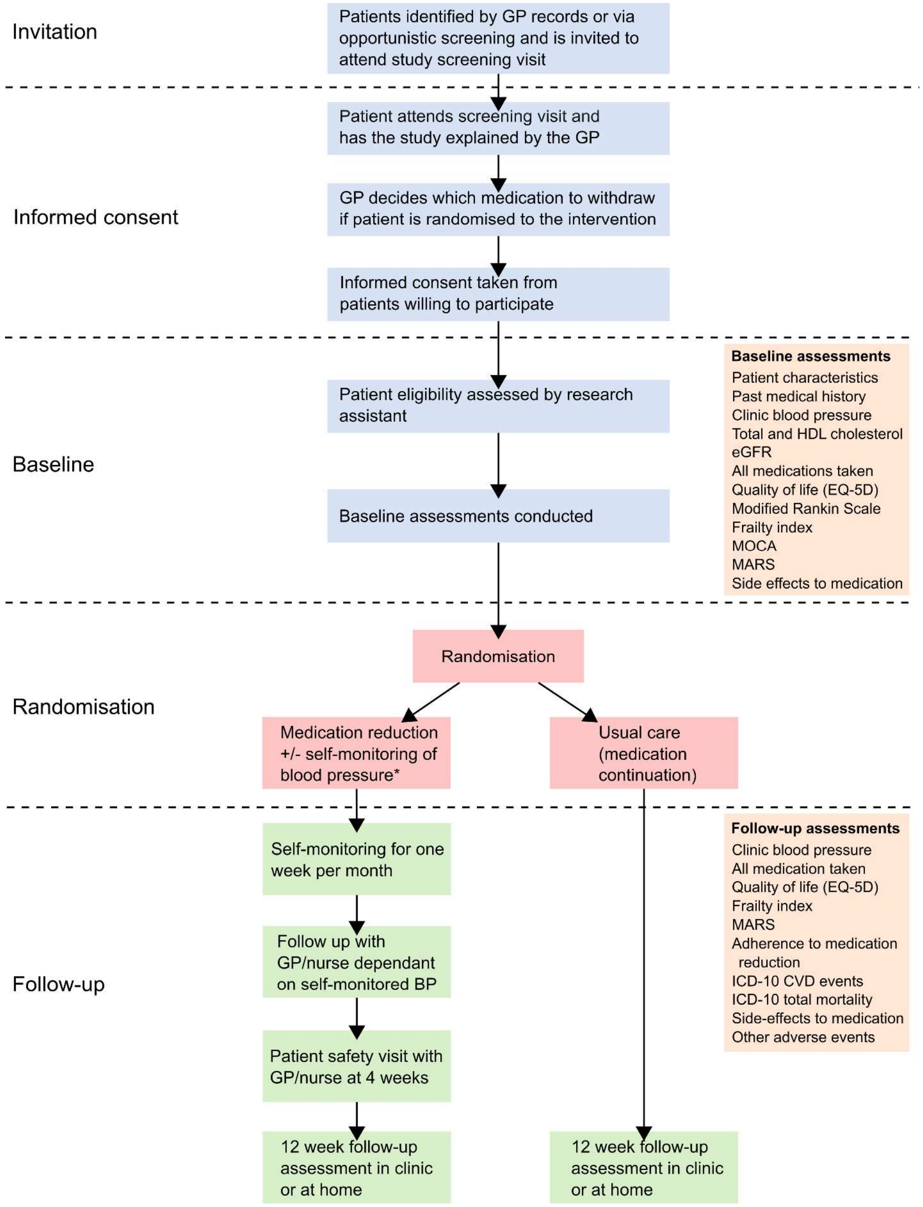
Invitation
Patients identified by GP records or via opportunistic screening and is invited to attend study screening visit
Informed consent
Patient attends screening visit and has the study explained by the GP
GP decides which medication to withdraw if patient is randomised to the intervention
Informed consent taken from patients willing to participate
Baseline
Patient eligibility assessed by research assistant
Baseline assessments conducted
Baseline assessments
Patient characteristics
Past medical history
Clinic blood pressure
Total and HDL cholesterol
eGFR
All medications taken
Quality of life (EQ-5D)
Modified Rankin Scale
Frailty index
MOCA
MARS
Side effects to medication
Randomisation
Randomisation
Medication reduction +/- self-monitoring of blood pressure*
Usual care (medication continuation)
Follow-up
Self-monitoring for one week per month
Follow up with GP/nurse dependant on self-monitored BP
Patient safety visit with GP/nurse at 4 weeks
12 week follow-up assessment in clinic or at home
12 week follow-up assessment in clinic or at home
Follow-up assessments
Clinic blood pressure
All medication taken
Quality of life (EQ-5D)
Frailty index
MARS
Adherence to medication reduction
ICD-10 CVD events
ICD-10 total mortality
Side-effects to medication
Other adverse events

Figure 1

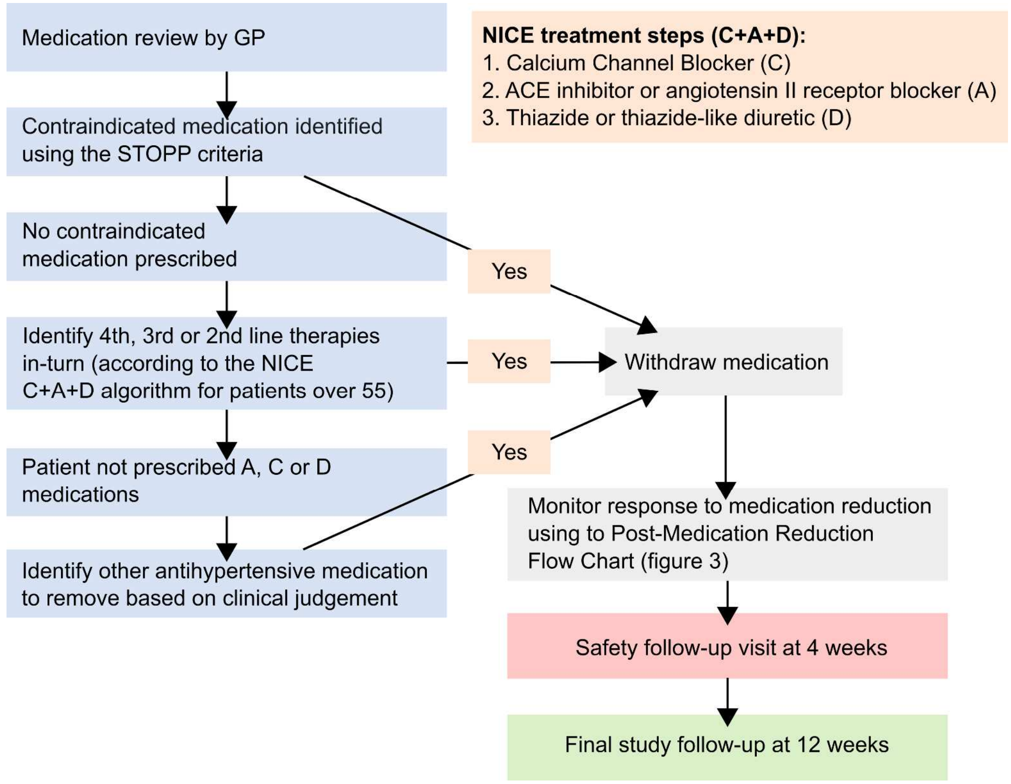

Figure 2

For peer review only - http://bmjopen.bmj.com/site/about/guidelines.xhtml

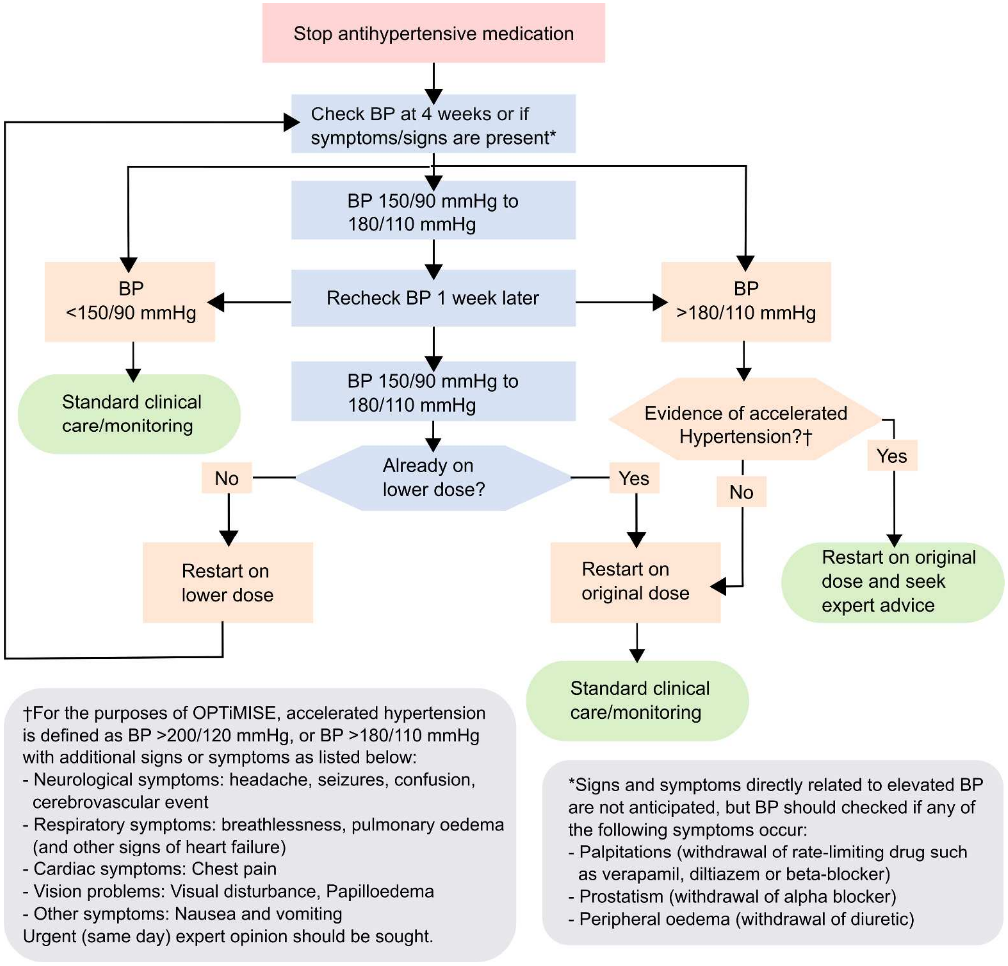

Figure 3

only

SPIRIT 2013 Checklist: Recommended items to address in a clinical trial protocol and related documents*

Section/item	Item No	Description	Page in protocol
**Administrative information**			
Title	1	Descriptive title identifying the study design, population, interventions, and, if applicable, trial acronym	1
Trial registration	2a	Trial identifier and registry name. If not yet registered, name of intended registry	2
	2b	All items from the World Health Organization Trial Registration Data Set	n/a
Protocol version	3	Date and version identifier	n/a
Funding	4	Sources and types of financial, material, and other support	12
Roles and responsibilities	5a	Names, affiliations, and roles of protocol contributors	1, 12
	5b	Name and contact information for the trial sponsor	1
	5c	Role of study sponsor and funders, if any, in study design; collection, management, analysis, and interpretation of data; writing of the report; and the decision to submit the report for publication, including whether they will have ultimate authority over any of these activities	12
	5d	Composition, roles, and responsibilities of the coordinating centre, steering committee, endpoint adjudication committee, data management team, and other individuals or groups overseeing the trial, if applicable (see Item 21a for data monitoring committee)	12
**Introduction**			
Background and rationale	6a	Description of research question and justification for undertaking the trial, including summary of relevant studies (published and unpublished) examining benefits and harms for each intervention	3
	6b	Explanation for choice of comparators	3
Objectives	7	Specific objectives or hypotheses	3-4
Trial design	8	Description of trial design including type of trial (eg, parallel group, crossover, factorial, single group), allocation ratio, and framework (eg, superiority, equivalence, noninferiority, exploratory)	4
**Methods: Participants, interventions, and outcomes**			
Study setting	9	Description of study settings (eg, community clinic, academic hospital) and list of countries where data will be collected. Reference to where list of study sites can be obtained	4
Eligibility criteria	10	Inclusion and exclusion criteria for participants. If applicable, eligibility criteria for study centres and individuals who will	4, table 1

For peer review only

		perform the interventions (eg, surgeons, psychotherapists)	
Interventions	11a	Interventions for each group with sufficient detail to allow replication, including how and when they will be administered	5
	11b	Criteria for discontinuing or modifying allocated interventions for a given trial participant (eg, drug dose change in response to harms, participant request, or improving/worsening disease)	Figure 3
	11c	Strategies to improve adherence to intervention protocols, and any procedures for monitoring adherence (eg, drug tablet return, laboratory tests)	6-7
	11d	Relevant concomitant care and interventions that are permitted or prohibited during the trial	7
Outcomes	12	Primary, secondary, and other outcomes, including the specific measurement variable (eg, systolic blood pressure), analysis metric (eg, change from baseline, final value, time to event), method of aggregation (eg, median, proportion), and time point for each outcome. Explanation of the clinical relevance of chosen efficacy and harm outcomes is strongly recommended	4-5
Participant timeline	13	Time schedule of enrolment, interventions (including any run-ins and washouts), assessments, and visits for participants. A schematic diagram is highly recommended (see Figure)	Figure 1
Sample size	14	Estimated number of participants needed to achieve study objectives and how it was determined, including clinical and statistical assumptions supporting any sample size calculations	8
Recruitment	15	Strategies for achieving adequate participant enrolment to reach target sample size	5
**Methods: Assignment of interventions (for controlled trials)**			
Allocation:			
Sequence generation	16a	Method of generating the allocation sequence (eg, computer-generated random numbers), and list of any factors for stratification. To reduce predictability of a random sequence, details of any planned restriction (eg, blocking) should be provided in a separate document that is unavailable to those who enrol participants or assign interventions	6
Allocation concealment mechanism	16b	Mechanism of implementing the allocation sequence (eg, central telephone; sequentially numbered, opaque, sealed envelopes), describing any steps to conceal the sequence until interventions are assigned	6
Implementation	16c	Who will generate the allocation sequence, who will enrol participants, and who will assign participants to interventions	6
Blinding (masking)	17a	Who will be blinded after assignment to interventions (eg, trial participants, care providers, outcome assessors, data analysts), and how	6

For peer review only

	17b	If blinded, circumstances under which unblinding is permissible, and procedure for revealing a participant's allocated intervention during the trial	6
**Methods: Data collection, management, and analysis**			
Data collection methods	18a	Plans for assessment and collection of outcome, baseline, and other trial data, including any related processes to promote data quality (eg, duplicate measurements, training of assessors) and a description of study instruments (eg, questionnaires, laboratory tests) along with their reliability and validity, if known. Reference to where data collection forms can be found, if not in the protocol	5-7
	18b	Plans to promote participant retention and complete follow-up, including list of any outcome data to be collected for participants who discontinue or deviate from intervention protocols	7
Data management	19	Plans for data entry, coding, security, and storage, including any related processes to promote data quality (eg, double data entry; range checks for data values). Reference to where details of data management procedures can be found, if not in the protocol	6
Statistical methods	20a	Statistical methods for analysing primary and secondary outcomes. Reference to where other details of the statistical analysis plan can be found, if not in the protocol	8
	20b	Methods for any additional analyses (eg, subgroup and adjusted analyses)	8
	20c	Definition of analysis population relating to protocol non-adherence (eg, as randomised analysis), and any statistical methods to handle missing data (eg, multiple imputation)	8
**Methods: Monitoring**			

For peer review only
